# 2-[2-(4-(trifluoromethyl)phenylamino)thiazol-4-yl]acetic acid (Activator-3) is a potent activator of AMPK

**DOI:** 10.1038/s41598-018-27974-1

**Published:** 2018-06-25

**Authors:** Navneet Bung, Sobhitha Surepalli, Sriram Seshadri, Sweta Patel, Saranya Peddasomayajula, Lalith Kumar Kummari, Sireesh T. Kumar, Phanithi Prakash Babu, Kishore V. L. Parsa, Rajamohan Reddy Poondra, Gopalakrishnan Bulusu, Parimal Misra

**Affiliations:** 1 TCS Innovation Labs-Hyderabad (Life Sciences Division), Tata Consultancy Services Limited, Madhapur, Hyderabad 500081 India; 2 Dr. Reddy’s Institute of Life Sciences, University of Hyderabad Campus, Gachibowli, Hyderabad 500 046 India; 3 Institute of Science, Nirma University, Ahmedabad, India; 4 Department of Biotechnology & Bioinformatics, School of Life Sciences, University of Hyderabad, Gachibowli, Hyderabad 500 046 India; 5 Institute for Molecular Bioscience, The University of Queensland, Brisbane, Queensland 4072 Australia; 6 Present Address: Australian Infectious Diseases Research Centre, School of Chemistry and Molecular Biosciences, The University of Queensland, Brisbane, Queensland 4072 Australia

## Abstract

AMPK is considered as a potential high value target for metabolic disorders. Here, we present the molecular modeling, *in vitro* and *in vivo* characterization of Activator-3, 2-[2-(4-(trifluoromethyl)phenylamino)thiazol-4-yl]acetic acid, an AMP mimetic and a potent pan-AMPK activator. Activator-3 and AMP likely share common activation mode for AMPK activation. Activator-3 enhanced AMPK phosphorylation by upstream kinase LKB1 and protected AMPK complex against dephosphorylation by PP2C. Molecular modeling analyses followed by *in vitro* mutant AMPK enzyme assays demonstrate that Activator-3 interacts with R70 and R152 of the CBS1 domain on AMPK γ subunit near AMP binding site. Activator-3 and C2, a recently described AMPK mimetic, bind differently in the γ subunit of AMPK. Activator-3 unlike C2 does not show cooperativity of AMPK activity in the presence of physiological concentration of ATP (2 mM). Activator-3 displays good pharmacokinetic profile in rat blood plasma with minimal brain penetration property. Oral treatment of High Sucrose Diet (HSD) fed diabetic rats with 10 mg/kg dose of Activator-3 once in a day for 30 days significantly enhanced glucose utilization, improved lipid profiles and reduced body weight, demonstrating that Activator-3 is a potent AMPK activator that can alleviate the negative metabolic impact of high sucrose diet in rat model.

## Introduction

Diabetes is a progressive disease of multiple metabolic disorders. The main cause is the absolute or relative deficiency of insulin. New agents with the properties of increasing insulin sensitivity, lowering glucose and having a beneficial effect on lipids, body weight and cardiovascular profiles will have potential health-care benefit. But the thiazolidinedione (PPARγ agonist) and glitazar (PPARα/γ dual agonist) saga highlights that the new agent should achieve these properties by modulating a different pathway^1–5^. AMP-activated Protein Kinase (AMPK) is a central energy regulator. Condition of energy depletion (low level of ATP) caused due to various stresses like prolonged exercise, heat shock, electrical stimulation of muscle or ischemia of muscle and inhibition of oxidative phosphorylation leads to the activation of AMPK resulting in replenishment of ATP and cellular energy balance by down regulating ATP consuming processes and accelerating ATP generation process^6–11^. Binding of AMP causes allosteric regulation of AMPK leading to change in the conformation and phosphorylation of the Thr^172^ at the kinase domain and protection from the entry and action of phosphatases, thereby preventing deactivation of AMPK^12,13^. In liver, activation of AMPK results in decreased production of plasma glucose, cholesterol, triglyceride and enhanced fatty acid oxidation^14,15^. In skeletal muscle, activation of AMPK is involved in the stimulation of glucose transport and fatty acid oxidation^16–20^. In adipose tissue, activated AMPK inhibits deposition of fat, but enhances breakdown and burning of stored fat, resulting in reduction of body weight^21–23^.

It is worth mentioning that AMPK activation by upstream kinase LKB1 mediates glucose homeostasis in liver and therapeutic effects of metformin by regulating CREB-PEPCK pathway^24^. Activated AMPK moderately decreases LDL by post translational modification of HMGCoA Reductase, a clinically validated target of statins^25^. Activation of AMPK regulates lipid metabolism by controlling the phosphorylation of the two isoforms of Acetyl-CoA Carboxylase (ACC). AMPK-mediated phosphorylation of ACC1 inhibits synthesis of free fatty acids through the modulation of intracellular concentration of Malonyl-CoA, a clinically validated lipid biomarker. On the other hand, phosphorylation of ACC2 accelerates the β-oxidation of the released free fatty acid from triglycerides^21,26^. Further, adiponectin, a natural AMPK activator, inhibits hypertrophic signaling in the myocardium through the activation of AMPK signaling^27^. Adiponectin may be a treatment of choice for hypertrophic cardiomyopathy associated with diabetes and other obesity-related disease^27^. These clinically well validated pathways have less commonality with PPAR-regulated pathways. Thus, AMPK has been identified as a possible diabetes/obesity target for many years. However, recent advances in structure, function, pathway analysis, HTS assays, studies with numerous tool compound activators and the demonstration that activation of AMPK is one of the mechanisms of action of the well-established and safe anti-diabetic agent metformin have stimulated a lot of interest in AMPK^28–37^.

AMPK is composed of three different subunits α, β and γ. In mammals, the heterotrimeric complexes combine catalytic α subunit (α1 or α2), with β (β1 or β2) and γ (γ1, γ2 or γ3) regulatory subunits encoded by separate genes yielding 12 heterotrimeric combination^38–43^. The α subunit contains a serine/threonine protein kinase catalytic domain in the *N*-terminal side, typical of the protein kinase super family^44^. The catalytic domain has a site of phosphorylation at residue Thr^172^ within the activation loop (T-loop), which is the key target site for AMPK activation by upstream kinase^44^. In the extreme C-terminus side, a region of ~150 amino acid residues is required for association with β and γ subunits, whereas the central part seems to possess an inhibitory function^45^.

AMPK can be directly activated by targeting α, β or γ subunits. The γ subunit has three isoforms (γ1, γ2 or γ3) and theoretically, selective modulator of each γ isoform should activate only four isozymes. AMP is a natural activator of AMPK and binds to the allosteric site of Cystein-β synthase (CBS) domains of different γ subunits (regulatory subunit) and thus indirectly promotes activity at the catalytic domain of α subunit^6^. The importance of AMP binding for AMPK activation is further demonstrated by mutations that localize to the CBS domains of the γ subunit in AMPK in Wolff-Parkinson-White Syndrome^46–49^. 5-aminoimidazole-4-carboxamide riboside (AICAR)-derived ZMP is an AMP analog and a potent AMPK activator that binds to the CBS domain of γ subunit^6^. Intravenous administration of AICAR reduces hepatic glucose output and inhibits whole body lipolysis in type 2 diabetic patients demonstrating proof of concept in human^30^. Though the trial has been conducted in very limited patients, AMPK has been validated as a target for diabetes in human. *In vivo*, AICAR is converted to ZMP^6^. But, AICAR derived ZMP is also a non-specific activator of AMP-binding proteins and long term use of this molecule may lead to unwanted side effect^50^. ZMP is a competitive binder of the natural metabolite AMP and binds to same CBS domains in AMPK, may inhibit AMP mediated physiological functions, which could be detrimental to the body^6,50^. There have been multiple allosteric activators of AMPK on the back of the first full length X-ray crystal structure published in 2013^51^. These include compound-7, PF-249, PF-739, 991 and MT-8722 targeted to the allosteric drug and metabolite (ADaM) site of AMPK^52–54^. The AdaM site is formed at the interface of carbohydrate-binding module (CBM) of the β subunit and the N-lobe of the α-kinase domain. The mechanism of action of A-769662, which binds to ADaM site, is one of the most studied aspects of AMPK research, the binding site and conformational changes leading to activation is well understood^34,51,55–57^. Most of the recent drug advancements in the AMPK field have been targeted toward the ADaM site, with the exception of Compound-2 (C2/C13), which is a γ subunit activaton^58–60^. A novel approach is to develop AMP mimetic small molecules as AMPK activators by targeting γ subunit that will have better selectivity and minimal side effects. Here, we report *in vitro* and *in vivo* characterization of Activator-3, a novel AMP mimetic small molecule and its probable binding sites in the CBS domain of the γ subunit of AMPK and its mechanism of action of AMPK activation.

## Results

### Effects of Activator-3 on AMPK activation in cell based assays

**Figure 1:**
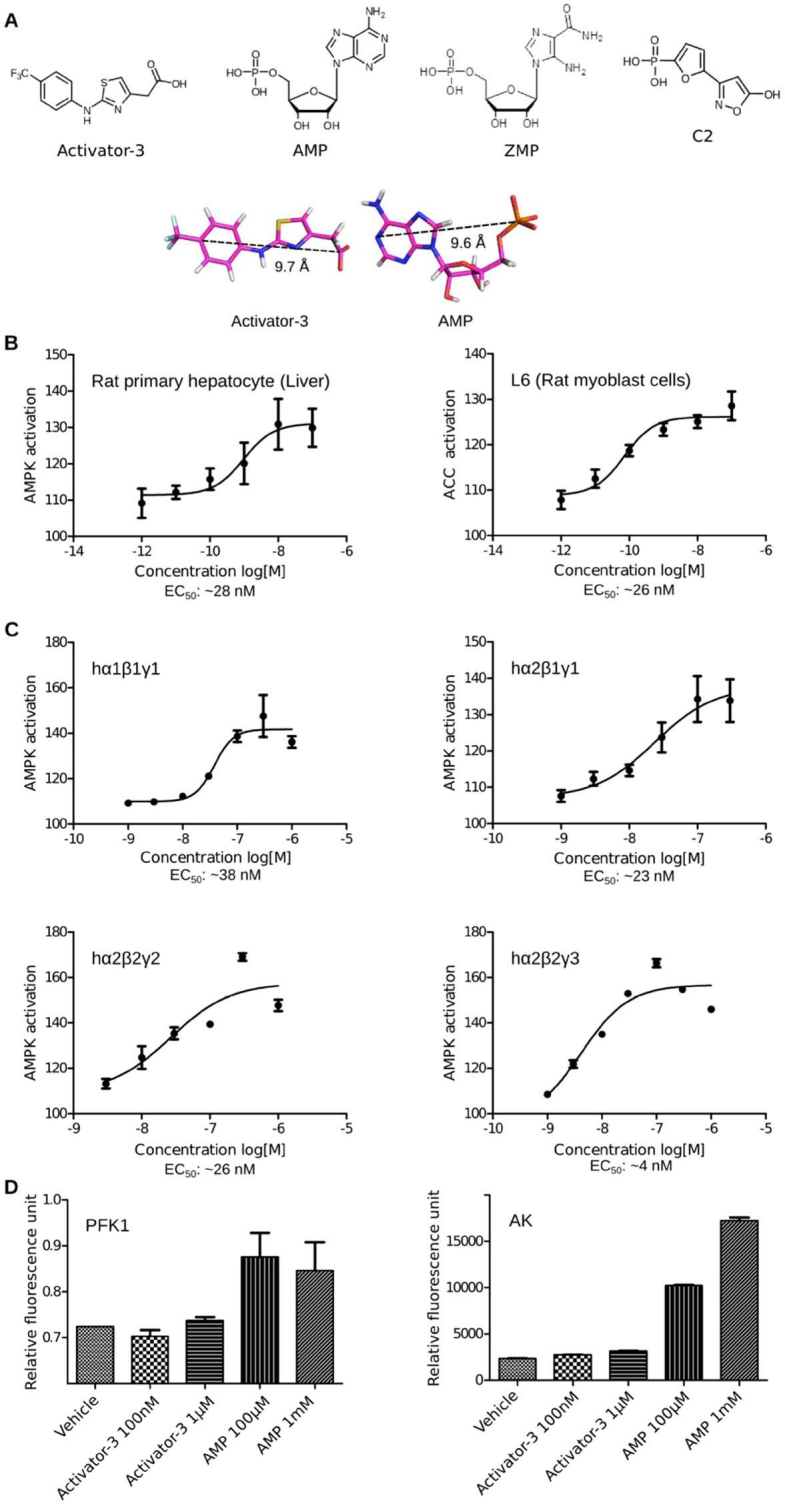
Activator-3, an AMPK mimetic is a potent pan-AMPK activator in cell based and cell free assays. (**A**) Chemical structures of Activator-3, AMP, ZMP and C2. Bottom panel: Rationale why Activator-3 may likely mimic AMP. (**B**) pAMPK based dose response curve of Activator-3 in primary rat hepatocytes (left panel) and rat L6 myoblasts (Right Panel). Results are expressed as the increase in activity relative to DMSO control and represent the mean ± SD for three independent experiments. (**C**) Human recombinant AMPK complexes (α1β1γ1, α2β1γ1, α2β2γ2 and α2β2γ3) expressed in baculo virus were assayed for allosteric activation by Activator-3. The representative four AMPK isozymes used for assays represent different combinations of all known reported isoforms like α1, α2, β1, β2, γ1, γ2 and γ3. EC_50_ values were calculated by plotting a non-linear curve of log [agonist] vs. response. Results are expressed as the increase in activity relative to DMSO control and represent the mean ± SD for three independent experiments. (**D**) Enzymes allosterically regulated by AMP (PFK1) or using AMP as a substrate (AK) were assayed in presence of AMP (≤1 mM) or Activator-3 (≤10 µM). Both the enzymes were unaffected by Activator-3. Results are representative of three independent experiments conducted using commercially available kits.

The structure of the natural and endogenous activator of AMPK, AMP and ZMP, an analog of AMP are shown in Fig. 1A along with the structures of C2, a γ subunit activator and Activator-3, an AMPK activator earlier reported by Dr. Reddy’s Laboratories Limited, Hyderabad, India (Fig. 1A)^61^. Activator-3 could be an AMP mimetic as the pharmacophoric distances between the aromatic moiety and the negative charge are similar (Fig. 1A).

A dose dependent response was observed in primary rat hepatocytes and rat myoblast L6 cells treated with varying concentrations of Activator-3 for the indicated time points. Activator-3 robustly activated AMPK with an EC_50_ of ~28 nM and ~32 nM for pAMPK in rat primary hepatocytes and L6 rat myoblasts, respectively (Fig. 1B). Similar EC_50_ were observed for pAMPK in HepG2 cells (Fig. S1A) and pACC in HepG2, primary rat hepatocytes and L6 cells as well (Supplementary Fig. S1B–D). These data suggest that Activator-3 is a potent nanomolar AMPK activator.

### Activator-3 directly binds and activates human recombinant AMPK complexes in cell free assays

Having observed the effect of Activator-3 on the activation of AMPK in cell based assays, we next examined whether Activator-3 could activate AMPK directly or not. For this, we have performed *in vitro* AMP kinase assays with purified human recombinant AMPK isozymes. The dose dependent direct stimulatory effect of Activator-3 on *in vitro* activation of recombinant human AMPK isozymes (α1β1γ1, α2β1γ1, α2β2γ2 and α2β2γ3) in the absence of AMP was detected by anti-phospho-ACC (Ser^79^) ELISA method. The activity of recombinant human AMPK (α1β1γ1, α2β1γ1, α2β2γ2 and α2β2γ3) in the presence of vehicle was considered as 100%. The EC_50_ of Activator-3 for the activation of α1β1γ1, α2β1γ1 and α2β2γ2 isozymes were similar. However, the activity of Activator-3 against α2β2γ3 was marginally higher (Fig. 1C). This data suggests that Activator-3 is a potent pan-AMPK activator *in vitro*.

### Effect of Activator-3 on other AMP- regulated enzymes and protein kinases

AMP is known to allosterically modulate several enzymes: 6-phos-phofructo-1 kinase (PFK1), gluconeogenic enzyme fructose-1,6-bisphosphatase-1 (FBP1) and phosphorylase b. Further, enzymes such as AMP deaminase-1, Adenylate kinase and 5′-nucleotidase use AMP as a substrate^62^. Thus, to examine the specificity of Activator-3 for AMPK, we have tested the activity of AMP and Activator-3 against PFK1 and Adenylate kinase using commercially available kits. As expected, AMP activated PFK1 at both the concentrations of 100 µM and 1 mM but Activator-3 had no effect on PFK1 even at 10 µM (>330 fold than EC_50_ of Activator-3 for AMPK; Fig. 1D). Moreover, we have tested the effect of Activator-3 on Adenylate kinase which uses AMP as a substrate. Adenylate kinase was minimally affected by 1 µM Activator-3 (Fig. 1D).

To determine whether Activator-3 affects the activity of any other protein kinases, we screened it in cell-free assays against a panel of 100 protein kinases (Kinexus, Canada). Majority of the protein kinases tested including several members of the AMPK-related upstream kinases such as TAK1 and LKB1 were not affected by 10 μM Activator-3 except CAMKK2 which exhibited 36% inhibition (Table S1 and S2). Three kinases, DDR1 (−79%), SRC (−71%) and ALK5 (−61%) were inhibited by Activator-3 by more than 50% at 10 µM. Further, four other kinases, LRRK2 (−34%), PAK1 (−42%), ROR2 (−35%) and PRK1 (−36%) were inhibited by Activator-3 by approximately 40% at 10 µM which is >330 fold higher than EC_50_ for AMPK activation in cell-based assays (Table S1). Taken together, these results suggest that Activator-3 is a rather specific AMPK activator.

### Activator-3 and AMP share common AMPK activation mode

**Figure 2:**
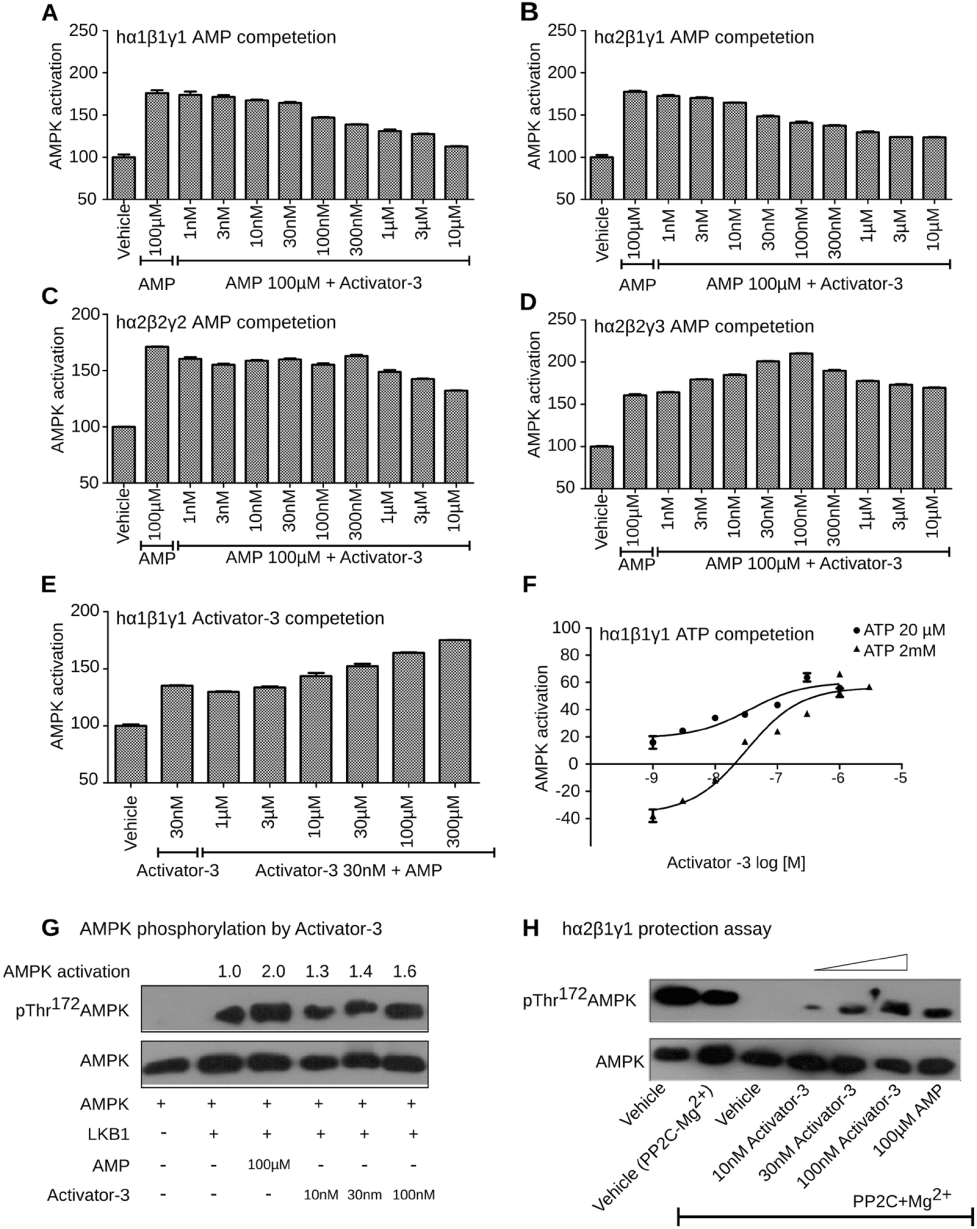
Activator-3 and AMP share common activation mode for AMPK activation and Activator-3 enhances AMPK phosphorylation by upstream kinase LKB1 and protects AMPK complex from PP2C mediated dephosphorylation. (**A**–**D**) Recombinant human AMPK α1β1γ1 (**A**), AMPK α2β1γ1 (**B**), AMPK α2β2γ2 (**C**) or AMPK α2β2γ3 (**D**) were assayed in the presence of AMP (100 µM) alone and AMP (100 µM) and increasing concentration of Activator-3 (0–10 µM). Results are expressed as percentage increase of AMPK activity relative to DMSO control and represent the mean ±SD for three independent experiments. (**E**) Recombinant human AMPK α1β1γ1 was assayed in the presence of Activator-3 (30 nM; ~EC_50_ concentration *in vitro* kinase assay) alone and Activator-3 (30 nM) and increasing concentrations of AMP (0–300 µM). Results are expressed as percentage increase of AMPK activity relative to DMSO control and represent the mean ± SD for three independent experiments. (**F**) Recombinant human AMPK α1β1γ1 was assayed in the presence of low concentration of ATP (20 µM) and high and physiological concentration of ATP (2 mM) and increasing concentration of Activator-3 (0–1 µM). Results are expressed as percentage change of AMPK activity relative to DMSO control (set as 100%). Results represent the mean ± SD for three independent experiments. (**G**) Recombinant AMPK complex was purified from HEK-293T cells by transient overexpression of the indicated construct as indicated in methods and assayed in presence of LKB1 alone or in presence of LKB1 and 100 µM AMP or increasing concentration of Activator-3 (10–100 nM) and the representative blot was quantified. (**H**) The effects of Activator-3 and AMP on dephosphorylation and inactivation of human recombinant AMPK α2β1γ1 by PP2C. Assays were performed either using vehicle (pAMPK alone) or with PP2C or vehicle with PP2C + Mg^2+^ or increasing concentration of Activator-3 (10–100 nM) or 100 µM AMP. The blots were probed with indicated antibodies.

To understand whether AMP and Activator-3 share a common binding and activation mode on the γ subunits, two different sets of experiments were performed. 1) AMP competition assay using different AMPK isozymes with Activator-3 concentration ranging from 1 nM to 10 μM and maintaining AMP concentration at 100 µM 2) Varied the AMP concentration (1 µM to 300 µM) keeping Activator-3 concentration at 30 nM. In the first set of experiments, Activator-3 antagonized AMP-dependent AMPK activation, reducing the activity of human recombinant AMPK isozymes α1β1γ1 and α2β1γ1 stimulated by 100 µM AMP by ~50% (Fig. 2A,B). The isozyme α2β2γ2 showed minimal decrease in the activity (Fig. 2C). Surprisingly, co-treatment of α2β2γ3 isozyme with Activator-3 and AMP increased AMPK activity >30% than 100 µM AMP alone (Fig. 2D). In the second set of experiments, we have observed that increasing concentration of AMP led to progressive enhancement of α1β1γ1 activity compared to the stimulation by 30 nM of Activator-3 alone (Fig. 2E). The above data indicate that AMP and Activator-3 may likely share a common binding site on the γ subunit or may function through a similar transduction mechanism as hypothesized.

ATP concentration plays an important role in the regulation of AMPK and AMP dependence^63^. Thus, to examine the effect of ATP on Activator-3 mediated AMPK activation, we have studied activation of human recombinant α1β1γ1 complex by Activator-3 under two different concentrations of ATP: low (20 µM) and high (2 mM; physiological). While 2 mM ATP inhibited AMPK activity at low concentrations of Activator-3, the inhibitory effects of ATP were reduced at high concentrations of Activator-3 (Fig. 2F) suggesting that Activator-3 and ATP (like AMP/ATP) may compete at the γ subunit allosteric sites and Activator-3 similar to AMP does not exhibit co-operative binding at high concentration of ATP^63^.

### Activator-3 enhances AMPK phosphorylation by upstream kinase, LKB1, and protects AMPK complex against dephosphorylation by PP2C

AMP allosterically activates AMPK and enhances the phosphorylation of pThr^172^ AMPKα by upstream kinases, a key control point of the overall activation mechanism of AMPK^63^. As Activator-3 is an AMP mimetic, we speculated that Activator-3 may show similar effect. To test this, recombinant AMPK complex was purified from HEK-293T cells overexpressing SFB tagged AMPK γ1 subunit and activity was studied in the presence of LKB1 alone or in the presence of LKB1 and 100 µM AMP or different concentrations of Activator-3 (10–100 nM). This analysis showed that similar to AMP, Activator-3 may also modestly enhance the LKB1-mediated phosphorylation of AMPK (Fig. 2G).

It is well documented that AMP activates AMPK allosterically by restricting the access to phosphatase and thereby conferring protection from dephosphorylation^63^. Activator-3, being a mimetic of AMP, was tested whether it is able to protect AMPK from dephosphorylation or not. Recombinant human AMPK α2β1γ1 isozyme was incubated with protein phosphatase PP2C in the presence or absence of AMP (100 µM) and increasing concentrations of Activator-3 (10–100 nM) and observed that similar to AMP, Activator-3 provided protection against PP2C mediated dephosphorylation of Thr^172^ AMPK in a dose dependent manner (Fig. 2H).

### Modeling AMPK structure

From *in vitro* cell based and cell free assays, it was concluded that Activator-3 is a potent activator of AMPK at nanomolar concentration. It is imperative to understand the binding mode of Activator-3. *In silico* methods such as molecular modeling, docking and dynamics were used to identify the binding pocket for Activator-3 molecule. For assembling α1, β1 and γ1 subunits, multiple template homology modeling in Modeler was used. The heterotrimeric homology model developed was energy minimized followed by multistep MD simulation.

### Molecular dynamics simulation of AMPK

**Figure 3:**
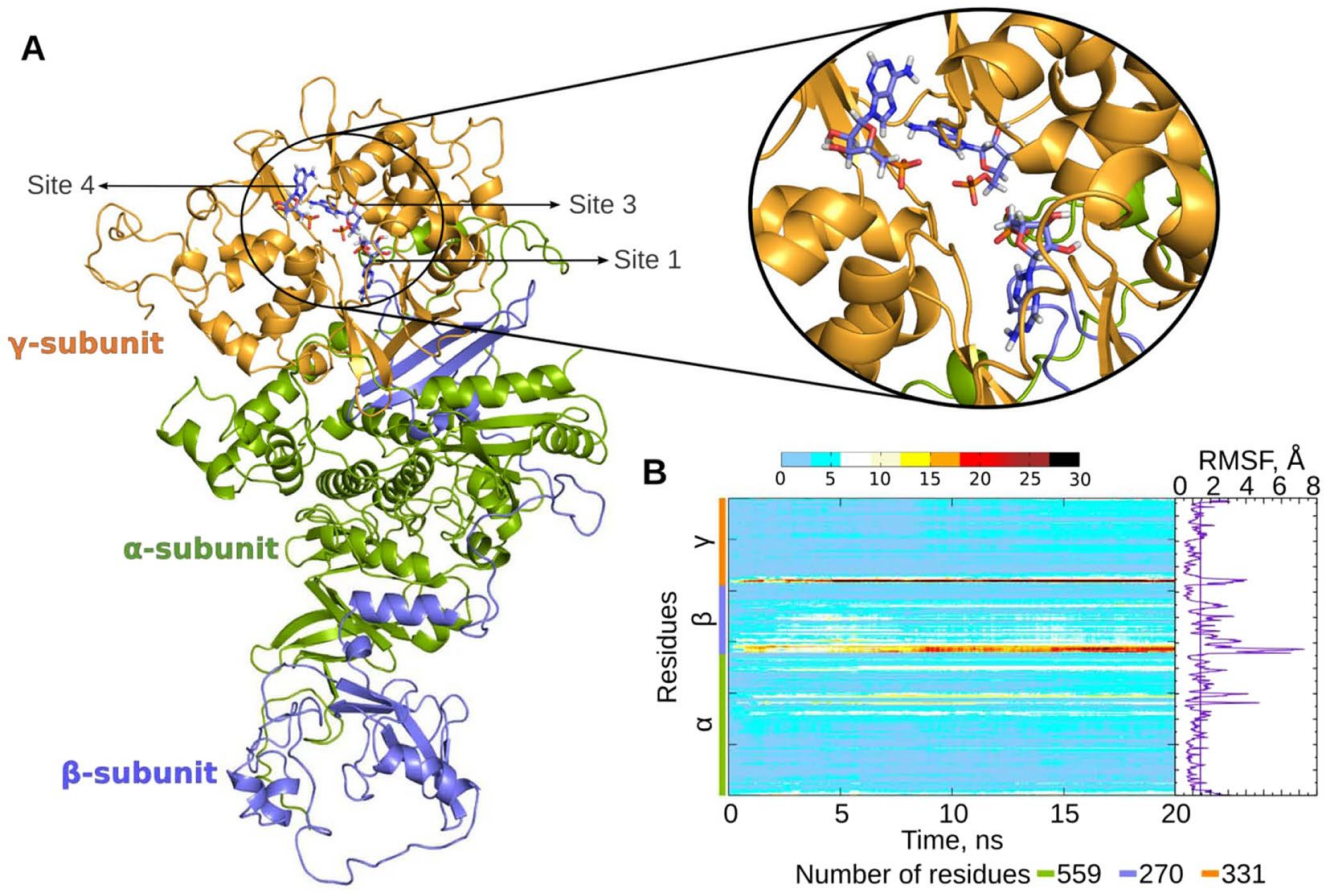
Molecular modelling of AMPK. (**A**) Complete structure of AMPK heterotrimeric complex with co-crystallized AMP molecules. The missing residues from the incomplete crystallographic structures were homology modeled. α, β and γ subunits are shown in green, blue and gold colors respectively. AMP molecules in the complex are shown in blue sticks. Inset shows a closer view of the three AMP bound sites in the γ subunit; (**B**) Heatmap showing residue-wise RMSD contribution of AMPK across the trajectory. The color code in the heatmap plot represents the RMSD value for each of the amino acids at a given time-frame. Root mean square fluctuations (RMSF) of the Cα atoms in the AMPK heterotrimeric complex are also shown. The vertical line in the RMSF plot represents the mean RMSF of the complex.

The energy-minimized structure was further refined by subjecting it for 20 ns MD simulation (Fig. 3A). The root mean square deviation (RMSD) of the protein backbone for each of the subunits with reference to the energy minimized AMPK structure is shown in Supplementary Fig. S2A.

The α subunit showed a minimal change in RMSD (approx. 4 Å) when compared to other subunits (Supplementary Fig. S2B). The β subunit showed maximum change in RMSD (approx. 7 Å) due to the long loop regions that connect its two segments (Supplementary Fig. S2C). Also, the γ subunit showed high deviation during the initial 10 ns of MD simulation due to the movement of N- and C- terminal regions (Supplementary Fig. S2D). Residue wise RMSD contribution is shown as a heatmap (Fig. 3B), which revealed maximum deviation for residues 1–40 (N-terminal of β subunit), 260–269 (C-terminal of β-subunit) and 1–20 (N-terminal of γ subunit).

From the RMSF plot, the maximum fluctuations were observed in most parts of β subunit (Fig. 3B). The fluctuations in the N- and C- termini of various subunits were in accordance with the observations made from the heat map calculation (Fig. 3B). Most of the secondary structural elements were stable throughout the simulation (Supplementary Fig. S2E).

The structure in the MD trajectory that showed least RMSD to the energy minimized cartesian-averaged structure was chosen as the representative structure. The representative structure was evaluated for goodness of model by PROCHECK. Based on the distribution of the dihedral angles phi and psi, 889 residues (85.8%) were in the most favored region of the Ramachandran map, whereas only 6 residues (0.6%) were in the disallowed region (Supplementary Table S3).

### Docking of Activator-3 in γ subunit

#### Finding new sites in the γ subunit

All the co-crystallized small organic molecules in the AMPK structure (PDB: 4CFF)^51^ were removed before docking. Four sites were identified for docking using sitemap tool (Schrodinger Software, Germany), out of which three were the existing sites, where an AMP molecule is bound in the AMPK crystal structures (PDB: 4CFF, 4CFE). The AMP molecule is bound at CBS1, CBS3 and CBS4 domains termed as sites 1, 3 and 4 in the current study. Additionally, a site at the interface of the four CBS domains of γ subunit (Site i) was chosen for docking studies.

#### Docking Activator-3, ZMP and AMP molecules

**Figure 4:**
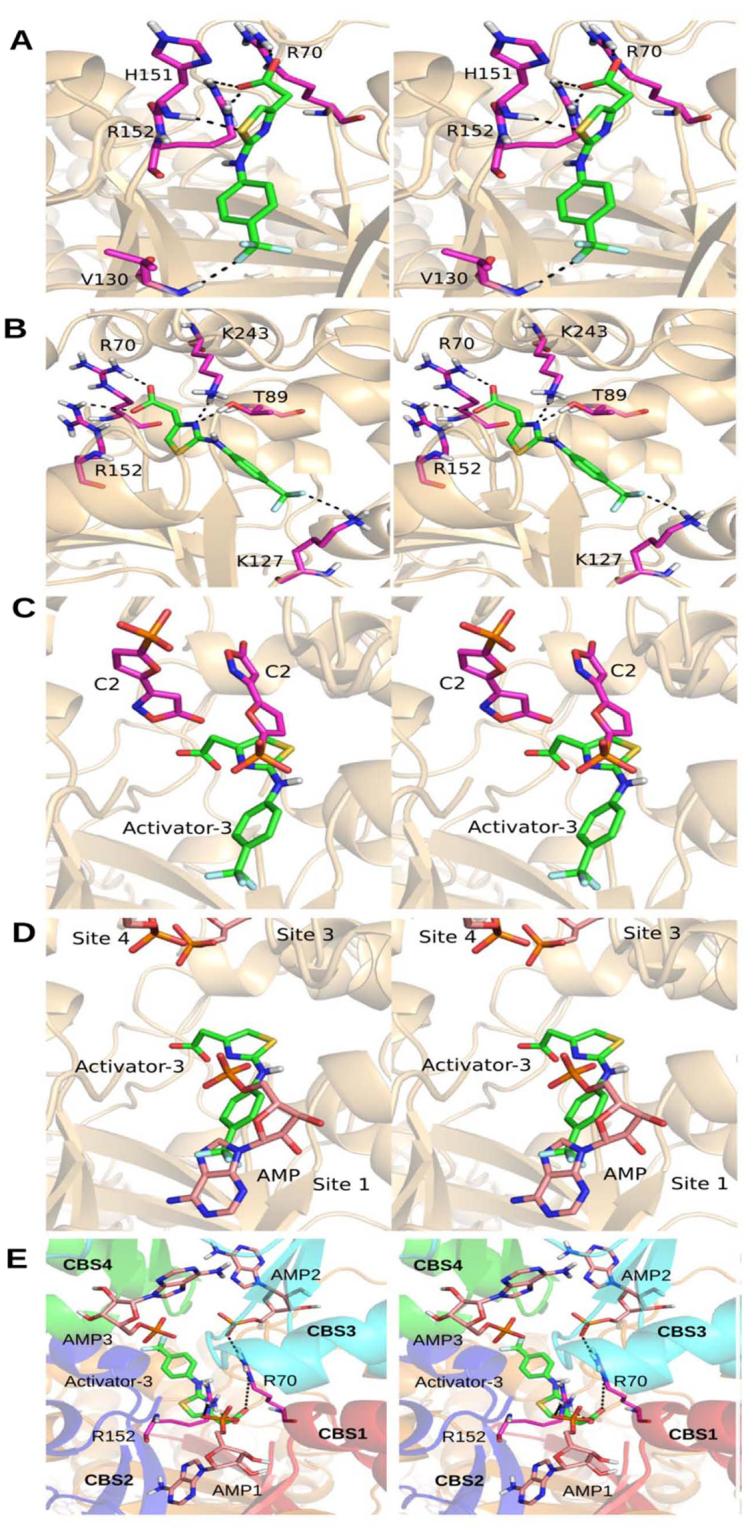
Docking studies of Activator-3 on AMPK. (**A**–**B**) AMPK-Activator-3 docked complexes at Site 1 (**A**) and Site i (**B**; Center of four CBS domains); considered for the current study. Activator-3 and interacting residues are shown in green and magenta sticks respectively. (**C**–**D**) Comparison of binding pose of Activator-3 with C2 (**C**) and AMP (**D**). (**E)** Activator-3 docked at the center of four CBS domains in presence of AMP (pink sticks) at all the three sites known from the crystal structure (PDB ID: 4CFF). In presence of AMP, the Activator-3 (green sticks) is docked at other side of R70 and R152 residues (magenta sticks). CBS domains 1, 2, 3 and 4 are shown in red, blue, cyan and green colors respectively. All the images are shown in stereo view.

AMP was used as the reference molecule to validate the docking protocol (Supplementary Fig. S3). Apart from Activator-3, ZMP was also used for the docking studies. AMP binds strongly (with high docking score) at each of the binding sites when compared to ZMP and Activator-3 molecule (Table 1). AMP docked at site 4 showed highest docking score, reflecting its non-exchangeable nature of binding to the CBS4 domain. At site 1, the carboxylate group of Activator-3 interacted with R70 and R152 residues while the -CF_3_ group interacted with the backbone of V130 residue (Fig. 4A). The Activator-3 at site 3 interacted with S242, E274 and R299 residues, while at site 4 it interacts with H151, A205, S214 and D317 residues (Fig. S4A,B). Though the center of the grid that was selected for sites 1 and i were different, the docking poses of Activator-3 molecule were similar at both the sites (Fig. 4A,D). At site i, apart from the interactions with R70 and R152, the Activator-3 interacted with K127 and K143 residues. Based on the docking score site 1 (−7.7) and site i (−7.4) could be the most probable sites for binding of Activator-3. The high score at sites 1 and i is due to the interaction of carboxylate group of Activator-3 with R70 and R152 residues, which act as an anchor for the binding of Activator-3.

**Table 1:** Docking score of AMP, ZMP and Activator-3 molecules at four sites that were identified in the γ subunit after removing all the co-crystallized ligands.

Docking Site	Molecule	Docking score
Site 1 (CBS1)	AMP	−12.2
	ZMP	−8.9
	Activator-3	−7.7
Site 3 (CBS3)	AMP	−14.1
	ZMP	−11.8
	Activator-3	−5.2
Site 4 (CBS4) non-exchangeable	AMP	−15.1
	ZMP	−9.6
	Activator-3	−4.3
Site I (At the interface of four CBS domain)	ZMP	−7.8
	AMP	−7.6
	Activator-3	−7.4
Docking in presence of AMP at Sites 1, 2 and 3	ZMP	−7.1
	AMP	−5.6
	Activator-3	−5.3

Docking calculation was also performed in presence of AMP molecule at sites 1, 2 and 3.

#### Comparison of binding of Activator-3 with C2 and AMP molecule

To compare the binding pose of Activator-3 to the C2 molecule, the X-ray crystal structure of AMPK (PDB Id: 4ZHX)^60^ was superposed to the representative structure of AMPK with docked Activator-3 molecule obtained from MD simulation (Fig. 4C). A careful examination of the binding pocket showed that the Activator-3 partially overlaps with C2 molecule present at the interface of four CBS domains (Fig. 4C). The carboxylate group of Activator-3 interacted with R70 and R152 residues, while the hydroxyl group of C2 molecule interacts with R299 in the γ subunit. The methyl carbon in Activator-3 molecule provides flexibility to the carboxylate group. It should be noted that in the crystal structure of AMPK with C2 bound there were two molecules of C2 per γ subunit^60^. However, precise stoichiometry of binding of Activator-3 could not be established based on docking experiments. The -CF3 group attached to the benzene ring of Activator-3 overlapped with the AMP molecule (Fig. 4D). While the other atoms of the Activator-3 overlapped with the C2 molecule (Fig. 4C,D).

#### Docking Activator-3, ZMP and AMP molecules in presence of co-crystallized AMP

Another docking calculation was performed, where AMP molecules at sites 1, 3 and 4 were retained. This helped to understand the effect of ligand binding cooperativity or the lack of it, during docking calculations. In the presence of AMP at three sites, Activator-3 is docked towards the other side of Bateman1 domain (interface of CBS 1 and 2 domains), which interacts with the regulatory interacting motif (RIM) 1 and 2 of the α subunit (Fig. 4E). Interestingly, the new docking pose of Activator-3 in presence of AMP molecules was also interacting with R70 and R152 residues, albeit with a decreased docking score (Fig. 4E). Based on the above experiment we can comment that in the absence of AMP molecules Activator-3 binds at site 1, near the CBS1 domain. Activator-3 binds on other side of Bateman 1 domain in the presence of AMP at all the three sites (Fig. 4E).

Based on the docking scores of with and without co-docked ligands, R70 and R152 are important for binding of Activator-3. These residues are being shared by AMP to allosterically bind AMPK γ subunit. In addition to this, a natural mutation R70Q in cardiac muscle cells constitutively activates AMPK and causes cardiomyopathy. Therefore, R70 and R152 were chosen and mutated to glycine for *in silico* and *in vitro* mutagenesis studies.

### Mutational studies

**Figure 5:**
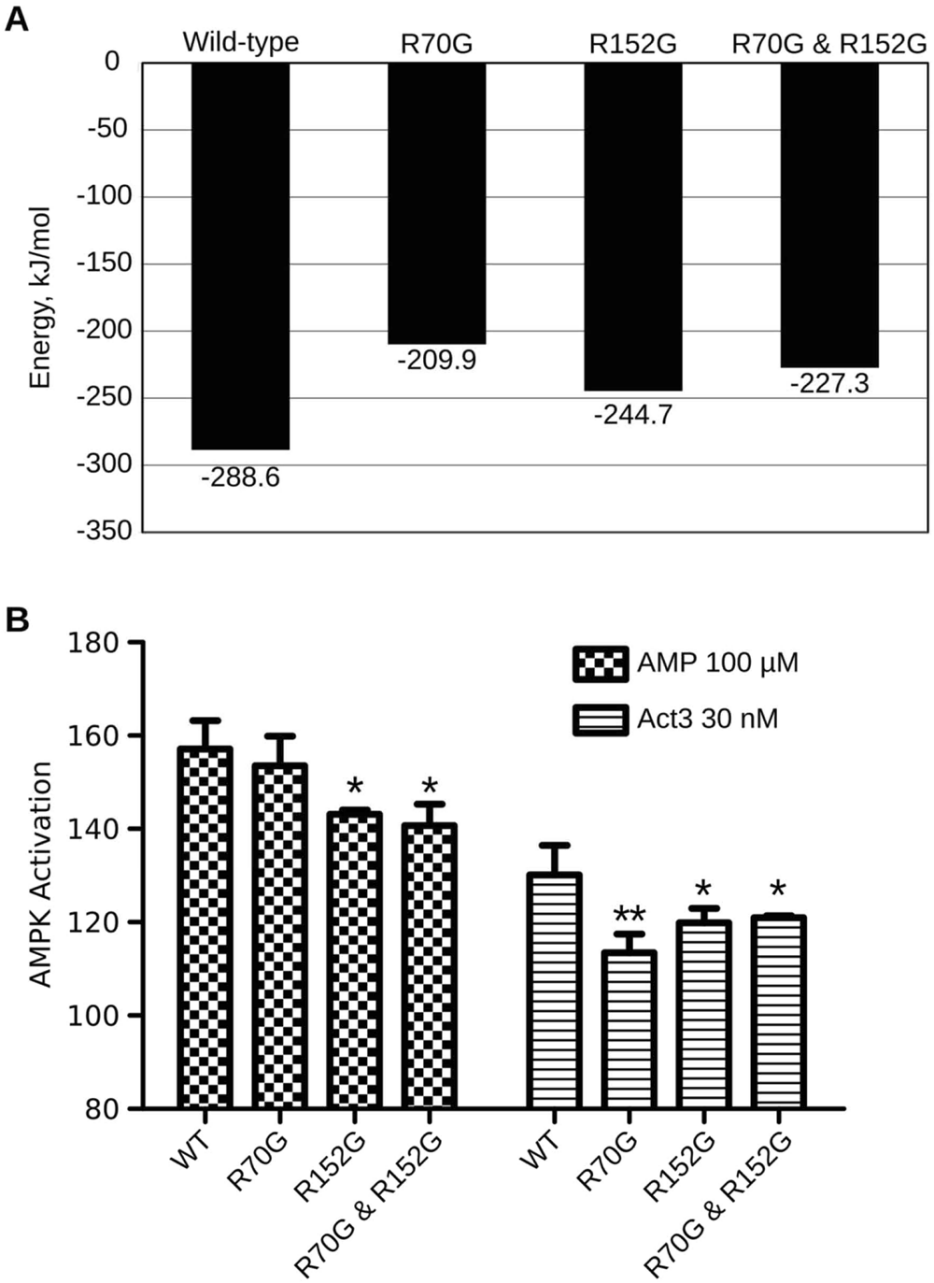
R70 and R152 amino acids γ1 subunit of AMPK are required for Activator-3 mediated AMPK activation. (**A**) The binding energy calculations of Activator-3 bound to wild type and mutant γ subunit of AMPK. (**B**) pACC based *in vitro* kinase activities of native human recombinant α1β1γ1 AMPK enzyme and its mutants using 100 µM AMP and 30 nM Activator-3. The activity shown in the above figure was normalized to vehicle control and wild type (WT) control group. Results are the representative of three independent experiments. Statistical analysis was performed using Bonferroni’s Multiple Comparison Test *p < 0.05, **p < 0.01.

To study the effect of mutations on ligand binding, three simulations were performed: two single residue mutations (R70G and R152G) and one double mutant (R70G and R152G; Fig. 5A). It is clear from the docking studies that Activator-3 interacts significantly weakly with the mutants compared to the wild type AMPK (Fig. S5).The strength of binding for each of the docked complexes was quantified using binding energy. The energy of Activator-3 binding to the γ subunit in wild type protein was −288 kJ/mol. Mutations of R70 and R152, responsible for the binding of Activator-3 in the wild-type protein, increased binding energy in all the mutant simulations (Fig. 5A) suggesting that residues R70 and R152 are probably crucial for the binding of Activator-3.

### Site directed mutagenesis study shows the importance of R70 and R152 residues located in the CBS1 domain of AMPK γ1 subunit for binding of Activator-3

*In silico* analyses showed that R70 and R152 of CBS1 domain of γ1 subunit are potentially important for the binding of Activator-3 to AMPK. Thus, we mutated the respective residues to glycine and overexpressed AMPK γ1 in HEK-293T cells and performed kinase assays with pull down samples (Fig. 5B and Supplementary Fig. S6A,B). The mutant AMPK proteins showed significant reduction in AMPK activity upon treatment with Activator-3 or AMP, revealing the importance of these residues in binding of Activator-3 to AMPK. However, R70G mutation did not impact the activation of AMPK by AMP (Fig. 5B).

### Activator-3 displays good pharmacokinetics profile in rat blood plasma with minimal brain penetration property

Male SD rats were orally administered 30 mg/Kg of Activator-3. Blood and brain samples were collected at specified time points and were analyzed for Activator-3 using a LC-MS/MS method^64–66^. Systemic exposure of Activator-3 in plasma (AUC) was ~978 µg*h/ml (~3.3 mM) in male SD rats after oral administration of 30 mg/Kg Activator-3 and t_1/2_ was 4.11 h post oral dosing (Supplementary Fig. S7). C_max_ in plasma was determined as ~109 µg/ml (~360 µM). The human plasma protein binding was ~80% (data not shown). No significant level of Activator-3 was detected in brain post oral administration (Supplementary Fig. S7). Systemic exposure of Activator-3 was very high in plasma compared to brain.

### Treatment of HSD fed insulin resistant diabetic rats with Activator-3 improves several metabolic parameters

**Figure 6:**
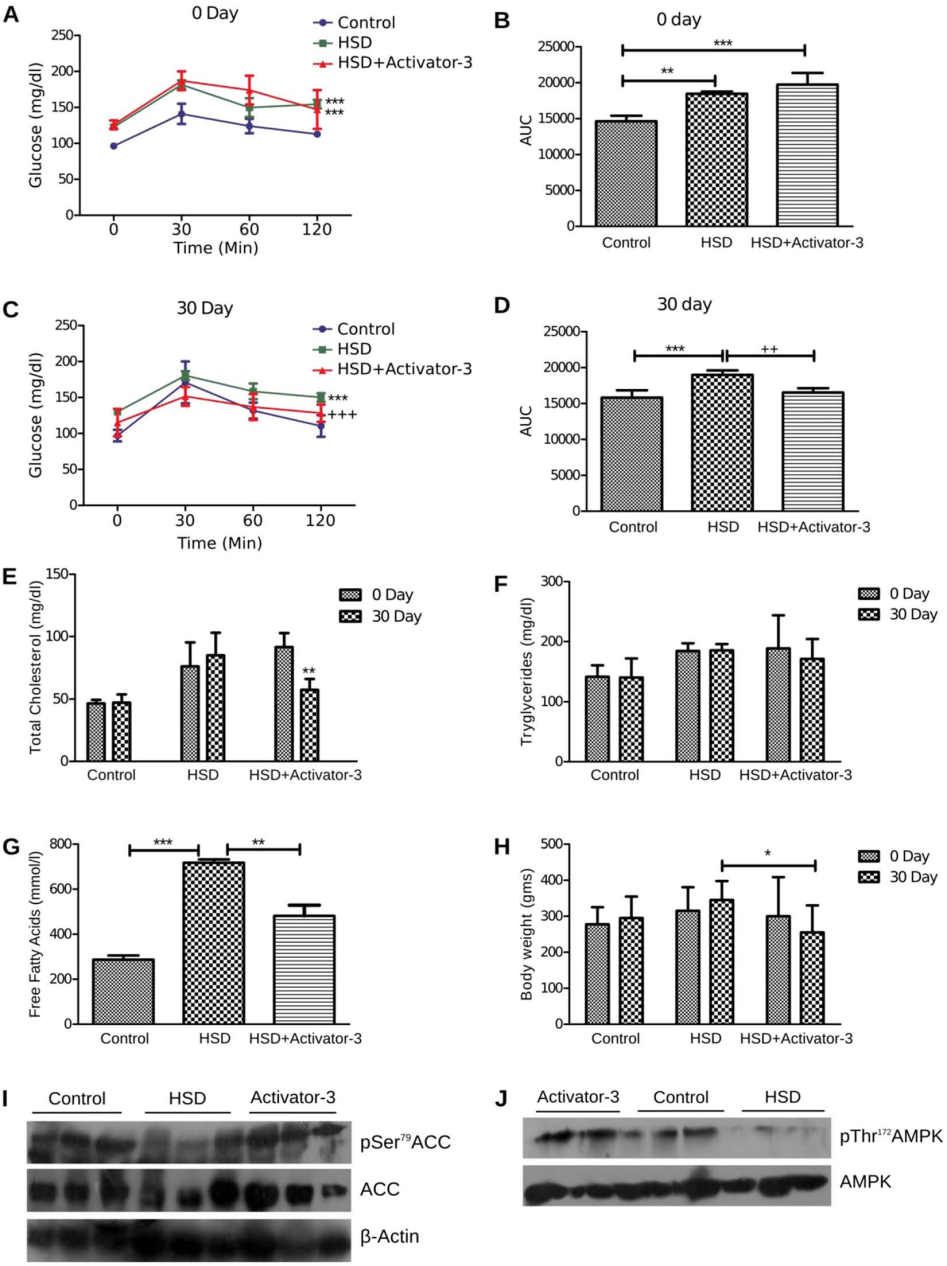
Treatment of HSD rats with Activator-3 improves metabolic health. (**A**–**D**) OGTT analysis of control and HSD rats on zero day (**A** and **B**) and 30 days (**C** and **D**). (**E**–**H**) Analysis of cholesterol (**E**), triglyceride (**F**) free fatty acid (**G**) and body weight (**H**) of control and HSD fed rats. Statistical analysis were performed using one way ANOVA followed by tukey’s post-hoc test. *p < 0.05, **p < 0.01, ***p < 0.001. (**I**,**J**). Western blot analysis of pAMPK, AMPK, pACC, ACC and β-actin of the soleus muscle tissue of normal, HSD fed rats treated with vehicle control or 10 mg/kg /day Activator-3 for 30 days.

The incremental changes in plasma glucose concentrations of rats following an oral glucose intake were observed in the groups. The glucose concentration of the HSD and HSD+Activator-3 group was significantly higher than those of control group at 30, 60 and 120 min on day zero (Fig. 6A,B). However on 30th day, the glucose concentration of the HSD group treated with Activator-3 was significantly reduced compared to HSD rats group (Fig. 6C,D) indicating increased insulin sensitivity in this group of animals (Fig. 6C,D).

At 0 day, plasma total cholesterol, triglyceride and free fatty acid levels were found to be significantly (*p* < 0.01) increased in HSD and HSD+Activator-3 group as compared to control group (Fig. 6E–G). On 30^th^ day, plasma total cholesterol and free fatty acid levels of HSD group treated with Activator-3 were significantly lower than HSD group alone (Fig. 6E–G). HSD feeding over 30 days slightly increased the body weight of animals as compared to the control diet animal groups (Fig. 6H). Activator-3 treatment of HSD group of animals for 30 days had significantly (p < 0.05) decreased HSD induced body weight gain (Fig. 6H). Further, the phosphorylation levels of AMPK α subunit and ACC were reduced in the soleus muscle of HSD fed rats and treatment of HSD fed rats with Activator-3 has restored the phosphorylation of both AMPK and ACC (Fig. 6I,J, Supplementary Fig. S8). These results indicate that the stimulation of the AMPK activation induces hepatic fatty acid oxidation, ketogenesis and leads to reduction of cholesterol synthesis, lipogenesis and adipocyte lipolysis in rats fed on a high sucrose diet.

## Discussion

Currently, there is no active pharmacological intervention available to stop or retard the progression of pre-diabetes to frank hyperglycemia. AMPK activation could be an important strategy to treat such patients who are in transition from pre-diabetic to diabetic stage; however several aspects have to be carefully considered. 1) Importantly, proof of concept studies in humans is not available. It is not known to what extent AMPK should be activated to achieve desired efficacy. 2) Further, as AMPK activation mimics a part of the physical exercise, it is not clear whether continuous activation or intermittent activation of AMPK is desirable. 3) Moreover, exercise modulates AMP/ATP ratio and thus activates AMPK. Thus, the AMPK activator should show complementary activity along with exercise. 4) It is argued that AMPK, a key energy sensor, controls post-translational modification and gene transcription. Whilst this makes it as a potent target, it also means that agonists will have significant effects on multiple pathways that may lead to adverse side effects. 5) Furthermore, inhibition of AMPK in hypothalamus reduced food intake while increase in the activity of AMPK in hypothalamus may increase food intake^67,68^. These facts make designing an allosteric activator of AMPK much more challenging.

Activator-3, exerted direct stimulatory effects on the activity of *in vitro* human recombinant AMPK isozymes namely α1β1γ1, α2β1γ1, α2β2γ2 and α2β2γ2 assays and moderate activation (30–40%) of AMPK was sufficient to exert *in vivo* activity in diabetic HSD rats. Furthermore, Activator-3 displayed good pharmacokinetics profile in blood plasma: t_1/2_ ~4 h, good bioavailability and no brain penetration property. Molecular modelling data suggested that R70 and R152 of the CBS1 domain of AMPK γ1 subunit are important for Activator-3 binding. Enzyme assays with recombinant human AMPK mutants demonstrated that Activator-3 indeed partially requires R70 and R152 of the CBS1 domain of γ subunit for its ability to activate AMPK and the same was observed with AMP. In case of AMP, R70G mutation has not resulted in a significant reduction in AMPK activation and the possible reason for this could be other compensating interactions of AMP with CBS domain of AMPK γ1. R70 of γ1, R302 of γ2 and R200 of γ3 subunits are similarly conserved amino acids located in their respective CBS domains of AMPK^47–50,69^. γ1 R70Q mutation causes a marked increase in AMPK activity^69^. Human mutation (R302Q) in the γ2 subunit encoded by PRKA γ2 results in chronic AMPK activation and causes cardiomyopathy characterized by undesirable effects like hypertrophy and glycogen storage^46–49^. The finding of increased glycogen content in skeletal muscle of Hampshire pigs harboring an R200Q variant in PPKAγ3, which is homologous to R302Q PRKAγ2 mutation in man, gives additional support to the role of R302 in the activation of AMPK^70^. These results demonstrate the importance of interaction of Activator-3 with R70 of γ1 for AMPK activation. However, Sanders *et al*. showed that due to R302Q mutation, γ2 subunit had lost its specificity towards both AMP and ATP^56^. This leads the authors to hypothesize that the γ2 subunit adopts a locked conformation as a result of the mutation and is no longer amenable for natural deactivation by phosphatases and/or ATP. In view of this, the authors believes that, in patients with no mutation in the γ2 subunit of AMPK, chronic activation would not lead to the above mentioned cardiac problem. This hypothesis is ably supported by the observations that adiponectin, a natural AMPK activator, inhibits hypertrophic signaling in the myocardium through activation of AMPK signaling and adiponectin have been use in the treatment of hypertrophic cardiomyopathy associated with diabetes and other obesity-related^27^. α2β2γ2 is the predominantly expressed AMPK isozyme in heart tissue. Activator-3 at saturated concentration (1 µM) showed EC_50_ of ~150% compared to basal activity and even in the presence of 100 µM AMP, the activity was minimally changed indicating that Activator-3 is a moderate AMPK activator. However, MK-8722 showed Emax of ~250% compared to basal activity indicating higher activation of α2β2γ2 isozyme^54^. Activation of AMPK should mimic the benefits of the bout of the exercise. The half-life of Activator-3 is ~4.0 h. Because of the short half-life and moderate Emax of activation, we speculate that Activator-3 may not be able to cause negative impact on the heart. Exogenous activators tend to bind in a site different to that of an endogenous activator (like AMP in AMPK) and stabilize the active conformation^52–54^. The purpose of utilizing computational tools is to find additional allosteric sites either in CBS domain or in the N-terminal of γ1 subunit of AMPK, because the mode of activation of Activator-3 is not completely understood. Molecular modelling study demonstrated that Activator-3 and AMP bind to and interact with few common and different amino acids located in the CBS domain of the AMPK γ subunit. The nature of the activator-binding pocket of Activator-3 suggests the involvement of additional sites of CBS domain of γ subunit of likely physiological importance in the regulation of AMPK. Importantly, these results offer new opportunities for the design of small molecule direct activators of AMPK which should act synergistically with AMP for treatment of metabolic disorders.

## Materials and Methods

### Homology modeling

The structure of complete α1β1γ1 isoform of human AMPK is not known (Supplementary Table S4). The available crystal structures (PDB ID: 4CFF and 4CFE) of human AMPK are incomplete and all the subunits of the heterotrimeric protein have missing residues (Supplementary Fig. S9)^51^. Therefore, the structures of all the three subunits were modeled independently and assembled based on the human α2β1γ1 isoform structure (4CFF).

#### α1 subunit

The complete primary sequence of human α1 subunit (Uniprot ID: Q13131) was retrieved from Uniprot protein knowledge base^71^. Sequence alignment with crystallized rat α1 subunit (4CFH_A) has shown three large gap regions corresponding to N-terminal (1–20), Auto-Inhibitory domain (AID) (292–331) and C-terminal (482–538). The structure of AID was modeled using 4F2L (PDB ID) as the template^72^. No significant templates could be identified for N- and C-terminal gap regions. So, complete sequence was submitted to I-TASSER^73^ for *ab inito* modeling.

#### β1 subunit

The primary sequence of human β1 subunit (Uniprot ID: Q9Y478) was retrieved from Uniprot protein knowledge base. The full length AMPK structures, 4CFE and 4CFF, have allosteric activating drugs 991 and A-769662 bound in the ADaM site^51^. The coordinates of CBM are reported in the 4CFF and 4CFE crystal structures. The coordinates were also reported for the 42 amino acid long loop region that joins two parts of β subunit. A homology model was generated using I-TASSER server, with 4CFF_B chain as user defined template.

#### γ1 subunit

The coordinates of γ1 subunit were available in PDB (PDB ID: 4CFF). The N-terminal missing residues (1–26) in the γ subunit were modeled using I-TASSER server.

### Heterotrimerization of AMPK

To generate the heterotrimeric complex of AMPK, the homology models of α1, β1 and γ1 subunits were superposed on the 4CFF AMPK structure. In AMPK heterotrimeric form, α and γ subunits interact head to head and a part of β subunit is placed in the cavity formed at the interface of α and γ subunits. After superposition of α1 and γ1 subunit on to 4CFF_A and 4CFF_E chains respectively the superposition of β1 subunit resulted in clashes with the α subunit. Modeller^74^ was used to re-model the β1 subunit into the heterotrimeric form by using the I-TASSER modeled β1 subunit as the template. To further refine the structure, energy minimization and molecular dynamic simulations were performed after docking the co-crystallized activators and inhibitors.

### Molecular dynamics

To check the stability of the homology modeled AMPK heterotrimeric structure explicit solvent molecular dynamics simulation was performed using Gromacs 4.5.5^59^ with amber99sb-ildn force field^75^. Force field parameters for the activator and inhibitor were obtained using antechamber tool^76^. The system was solvated in an octahedron box with a 9 Å layer of TIP3P model water molecules; protein charges were neutralized by adding sodium ions. The system was then energy minimized using steepest descent followed by NVT and NPT position restrained equilibration for 200 ps and 500 ps respectively^77^. A weight of 1000 kcal/mol Å^2^ was used to restrain the heavy atoms during the equilibration step. The temperature was gradually raised from 0 K to 300 K at 3 K/ps. Bond lengths were constrained using LINCS algorithm. Periodic boundary conditions were employed to minimize edge effects and the electrostatic computations were done using Particle Mesh Ewald^78^ with interpolation order of 4, tolerance of 1e-5 and Fourier spacing of 1.6 Å. Production run of 20 ns was performed on the ligands docked-AMPK complex using NPT ensemble. The goodness of the modelled structure was evaluated using PROCHECK^79^.

### Docking of Activator-3 in the γ subunit

AMP, ZMP and Activator-3 were minimized using the ligprep module of Schrodinger. Ligands were docked using extra precision (XP) mode^80^. Post docking, minimization was performed on each of the docked complexes and a strain correction term was added to the final score. 10 poses were generated for each of the docking calculations. The structure with best docking score was selected for further analyses. To check for persistent interactions during the docking studies, the enzyme-activator complexes were minimized and subjected to molecular dynamic simulations using the protocol mentioned above. Production run of 10 ns was performed on the Activator-3 docked AMPK complex using NPT ensemble. Binding energies for the trajectories obtained from MD simulations for each of the complexes were calculated using g_mmpbsa^81^ module of Gromacs^82^.

### Cell culture materials and reagents

Cell lines HepG2 (ATCC Cat. No. HB-8065) and L6 myoblasts (ATCC Cat. No. CRL-1458) were cultured in DMEM containing 10% FBS (Fetal Bovine Serum) at 37 °C and 5% CO_2_. When the cells reached confluence, cells were trypsinzed with 0.05% Trypsin for 5 min. Activity of trypsin was inhibited by addition of equal volume of 10% FBS + DMEM and cells were centrifuged at 200 g for 3 min. Pellet was re-suspended in 1 ml of complete medium and cells were counted using hemocytometer.

### Primary hepatocytes isolation

Primary rat hepatocytes were isolated by collagenase (type4, Invitrogen: Cat# 17104-019) digestion as described previously^83^. Digested cells were suspended in DMEM supplemented with 10% FBS. Cells were seeded into 96-well plate (20,000 cells per well) pre-coated with collagen (2 mg/ml). As the cells were metabolically active, medium was changed after cells have attached and allowed to incubate overnight before performing cell based assay.

### Cell based ELISA

Approximately 40,000 cells per well were seeded into a 96 well plate (Biofil, cat# TCP011096) and incubated overnight at 37 °C and 5% CO_2_. After the seeded cells reached 80% confluence, cells were treated with Activator-3 with different working concentrations and 500 μM AICAR (Caymen chemicals, cat# 10010241-10) and incubated for 30 min to 2 h (depending upon the primary antibody used) at 37 °C and 5% CO_2_. Medium was aspirated and fixed with 4% of formaldehyde solution (diluted in 1X PBS) incubated for 1 h at 4 °C on shaker. Cells were washed with tween 20 (diluted in 1X PBS) on shaker for 5 minutes at room temperature (3 times). To quench the endogenous peroxidase activity cells were incubated with 100 µl per well of quenching solution (1% H_2_O_2_ in 1X PBS) in dark. Plate was incubated for 45 minutes at 37 °C and 5% CO_2_. Subsequently plate was washed three times as mentioned above. A permeabilization step was done by adding 100 µl per well of 0.05% Triton–X–100 and incubated for 5 min on shaker at room temperature and washed 3 times appropriately. To avoid nonspecific binding of antibody, cells were blocked by adding 300 µl per well of 5% BSA and incubated for 1 h on shaker at room temperature followed by overnight incubation with pAMPK (Thr172)/pACC (Ser79) diluted in 5% BSA (1:1000 dilution; Cat#2531/3661 Cell Signaling technology). To quantify the bound primary antibody, cells were probed with a HRP conjugated secondary anti-rabbit antibody (1:2000 dilution, Bio-Rad; Cat# 170-6515) diluted in 1X PBST and incubated 2 h at RT on rocker. After 2 h incubation, plate was washed firmly with the washing solution for 3 times and subsequently added 1X TMB/H_2_O_2,_ (Bangalore Genei: 106035) in dark and incubated till the color is developed. This reaction is stopped by adding 2 N H_2_SO_4_ and plate was immediately read at 450 nm using UV–Vis Spectrophotometer.

### *In vitro* AMP kinase assay

*In vitro* kinase assay was done with recombinant human AMPK α1β1γ1, α2β1γ1, α2β2γ2 and α2β2γ3 isozymes (50 ng per well: SignalChem, Cat# P47-10H-05/P48-10H-05) either with Activator-3 or AMP or AMP and Activator-3 as described in different experiments. A high binding Enzyme Immuno Assay (EIA) 96-well plate (Sigma: Cat# CLS3590) was coated with Acetyl CoA Carboxylase 2 (ACC: 175–271 amino acids) peptide, a direct substrate for AMPK, 100 ng per well (1:1000 ratio, Cat# 12–491, Upstate biotechnology) and incubated overnight at 25 °C. Reaction mixture was incubated at 30 °C for 2 h and reaction was stopped by washing the plate with 0.05% Tween 20 followed by incubation with pACC primary antibody and rabbit secondary antibody as described in cell based ELISA section. Assay buffer is composed of 10 mM DTT, 100 mM magnesium acetate, 50 μM ATP and 10 mM HEPES.

### Phosphatase protection Assay

Human recombinant α2β1γ1 isozyme was incubated with Activator-3 or AMP for 30 min at 30 °C in 10 mM MgCl_2_, 10 mM DTT, 50 mM Tris-HCl pH 7.4 and 0.5% Triton-X-100 and was then incubated with human recombinant PP2C (Catalog # 12984-H08E: Sino Biologicals) for about 30 min. The reaction was stopped by adding SDS-loading dye immediately to the reaction mixtures followed by boiling the samples at 95 °C for 10′. These samples were run on a 10% SDS-PAGE and probed for AMPK and pAMPK (1:1000; Cell Signaling Technologies).

### Phosphorylation by Activator-3 mediated by LKB1

SFB (S-protein, Flag and Streptavidin binding peptide) tagged AMPK γ1 sub-unit was overexpressed in HEK-293T cells and precipitated with streptavidin beads. The precipitates were incubated with either Activator-3 or AMP in 10 mM Magnesium Acetate, 10 mM DTT, 50 mM Tris-HCl pH-7.4 and 50 µM ATP for 30 min at 30 °C and subsequently human recombinant LKB1 (Catalog # AB119736: Abcam) was added and incubated under same conditions. This reaction was immediately stopped with SDS-loading dye and resolved on 10% SDS-PAGE and probed for AMPK and pAMPK (1:1000; Cell Signaling Technologies).

### PFK and AK activity assays using Activator-3

Effect of Activator-3 on AMP regulated enzymes such as Phospho Fructo Kinase1 (PFK1) (Catalog # K776-100: Biovision) and Adenylate Kinase (AK) (Catalog # K350–100: Biovision) was studied using the activity assay kits following manufacturer’s instructions.

### Site directed mutagenesis, transfection and pull down

R70 and R152 residues in AMPK γ1 subunit (pDONR223-PRKAG1: purchased from addgene, cat# plasmid#23718) are mutated to glycine using overlapping extension PCR. Subsequently, the mutants were transferred into a triple tagged destination vector (Streptavidin, Flag and Biotin; SFB-pDEST) by using gateway cloning technology. The mutant DNA was transfected into HEK-293T cells using PEI following standard procedures. After 4 h of transfection, media was changed to DMEM containing 10% FBS. Cells were lysed in TENNS lysis buffer (20 mM Tris pH:8.0, 100 mM NaCl, 1 mM EDTA, 10 mM Na_2_H_2_P_2_O_7_, 10 mM NaF, 10 mM Na_3_VO_4_, 0.5% NP40) and subjected to pull down using streptavidin beads followed by biotin elution. To protect the protein from dephosphorylation, cells were treated with 500 μM AICAR prior to lysis. This purified protein was estimated by using nanodrop and taken for *in vitro* kinase assay to check whether Activator-3 is binding to the mutant AMPK or not, and the activity of the mutant protein was assessed by comparing with the wild type protein activity.

### Western blotting

Approximately 100 mg of soleus muscle tissue was taken and lysed with RIPA lysis buffer. Lysates were resolved on 12% SDS–polyacrylamide gels and were transferred to PVDF membrane (Millipore, USA). Membrane was blocked with 5% nonfat milk in TRIS buffered saline (TBS; 10 mM TRIS (pH 8.0), 150 mM NaCl) for 1 hour and probed with primary antibody of interest: phospho-AMPK (Thr^172^) (1:1000; Cell Signaling Technology, USA), phospho-ACC (Ser^79^) (1:1000; Cell Signaling Technology, USA), total AMPK (1:1000; Cell Signaling Technology, USA), total ACC (1:1000; Cell Signaling Technology, USA), and total Actin (1:1000; Santa Cruz Biotechnology, USA). Subsequently, membranes were incubated with corresponding HRP-conjugated secondary antibodies. The chemiluminescence signals were captured on photographic films using an enhanced chemiluminescence (Thermo Scientific, USA).The loading controls of phospho-ACC and phospho-AMPK in Fig. 6J were probed in parallel gels.

### Statistical Analysis

Values were expressed as mean ± SD. For comparison between 2 groups, the unpaired Student’s t test was used. For comparisons of 2 or more groups Two-Way ANOVA was used followed by Bonferroni’s post-hoc analysis, *p* < 0.05 was considered as significant.

### Pharmacokinetic study in male rats

Male SD rats were orally administered with 30 mg/Kg of Activator-3. Blood and brain samples were collected at specified time points and were analyzed for Activator-3 using optimized LC-MS/MS method^64–66^.

### Animal treatment

Twelve weeks old healthy colony bred male Wistar rats, weighing about 150–200 grams were purchased from the animal research facility (Cadila Pharma limited, Ahmedabad, India) and maintained at the animal house of Institute of pharmacy (Nirma University, India). All the methods were carried out in accordance with the approved guidelines. Experimental protocol involving animals was reviewed and approved by the Animal Ethical Committee of Institute of Science, Nirma University, Ahmedabad, India (Protocol No. IS/FAC/14-1/021) Animals were maintained in polypropylene cages in a standard photoperiod (12 h light:12 h dark cycle) and temperature (27 ± 1 °C) controlled room with the provision of laboratory food (Gold Mohur feeds Ltd, New Delhi, India) and water *ad libitum*. Animals were segregated into three different experimental groups with 6 animals in each as follows: non-diabetic group with standard chow diet (control group, CD), Diabetic group with high sucrose diet (65%, treated group, HSD)^84^ and HSD with AMPK Activator-3 treated group (10 mg/kg body weight of animal). Post HSD induction animals were treated with AMPK Activator-3 for 30 days.

### Oral Glucose Tolerance Test (OGTT)

Animals were subjected to an OGTT at a beginning of the treatment (0 day) and a day before the end of the experiment (30 day). OGTT was performed after 12 h of the fasting. Briefly, after fasting, animals were given a glucose load (2 g/kg) orally. Blood samples were collected from the animal tail vein at 0 min (before glucose administration) and at 15, 30, 60, 90 and 120 min after glucose administration. Glucose concentration was determined using a CareSensN blood glucose monitor and glucose strips. Area under the curve for glucose (AUC_glucose_) was calculated using the trapezoidal rule.

### Blood collection

Approximately, 1 ml of the blood samples was collected from the retro-orbital plexus of the animals under the mild anesthesia using diethyl ether. Blood was then transferred to the microfuge tubes for separation of plasma and serum at 0 day (start of the treatment) and the last day of the experiment (30 day). Total cholesterol and triglycerides levels were estimated from plasma using a diagnostic kit from Accucare India Ltd according to the protocol mentioned by the manufacturer.

### FFAs and Insulin Estimation

Plasma FFA was estimated using chloroform and triethyalanomine extraction method by measuring the absorbance at 440 nm. The stearic acid was used as standard fatty acid and detailed protocol was performed as mentioned^85,86^. Fasting Insulin level from the serum samples were estimated using radioimmunoassay methods.

## Electronic supplementary material

Supplementary information
