# Supplementary material for: 2-[2-(4-(trifluoromethyl)phenylamino)thiazol-4-yl]acetic acid (Activator-3) is a potent activator of AMPK
Source: Sci Rep. 2018 Jun 25;8:9599. doi: 10.1038/s41598-018-27974-1 (PMC6018554; doi:10.1038/s41598-018-27974-1)
Supplement: Supplementary file 1 — Supplementary information [file 41598_2018_27974_MOESM1_ESM.pdf]

Supplementary tables and figures

2-[2-(4-(trifluoromethyl)phenylamino)thiazol-4-yl]acetic acid (Activator-3) is a potent activator of AMPK

Navneet Bung[1]+, Sobhitha Surepalli[2]+, Sriram Seshadri[3], Sweta Patel[3], Saranya Peddasomayajula[2], Lalith Kumar Kummari[2@#], Sireesh T Kumar[4], Phanithi Prakash Babu[4], Kishore V.L. Parsa[2], Rajamohan Reddy Poondra[2], Gopalakrishnan Bulusu[1,2]* and Parimal Misra[2]*

+Equal contributors

***Corresponding authors:** g.bulusu@tcs.com**;** parimalm@drils.org

Table S1: Activity change observed in the selected 100 protein kinases tested against Activator-3 at 10μM concentration.

No.	Protein Kinase	Control (CPM)	Test (CPM)	Difference Test-Control	% Activity	% Change
1	ABL1	574,777	561,149	13,628	98%	2%
2	AKT1	256,826	231,604	25,222	90%	10%
3	ASK1	63,215	65,142	(1,927)	103%	-3%
4	AURORA A	57,230	51,188	6,042	89%	11%
5	AXL	27,937	27,537	400	99%	1%
6	BMPR2	52,011	49,850	2,161	96%	4%
7	BRK	97,389	105,326	(7,937)	108%	-8%
8	BTK	19,951	19,215	736	96%	4%
9	BUB1B	5,894	5,416	478	92%	8%
10	CAMK1 beta	692,242	723,296	(31,054)	104%	-4%
11	CAMKK2	73,703	46,831	26,872	64%	36%
12	CASK	1,676	1,542	134	92%	8%
13	CDK1/CyclinA1	119,703	109,114	10,589	91%	9%
14	CDK2/CyclinA1	142,944	103,234	39,710	72%	28%
15	CDK4/CyclinD3	82,745	86,478	(3,733)	105%	-5%
16	CHK1	71,583	72,993	(1,410)	102%	-2%
17	CK1 alpha 1	72,832	66,138	6,694	91%	9%
18	c-KIT	35,085	36,179	(1,094)	103%	-3%
19	CLK1	230,212	243,270	(13,058)	106%	-6%
20	CSK	202,650	203,223	(573)	100%	0%
21	DAPK1	123,986	123,065	921	99%	1%
22	DCAMKL1	46,200	39,568	6,632	86%	14%
23	DDR1	14,327	3,006	11,321	21%	79%
24	DMPK	85,414	98,852	(13,438)	116%	-16%
25	DYRK1A	238,298	234,337	3,961	98%	2%
26	EEF2K	150,251	165,305	(15,054)	110%	-10%
27	EIF2AK4(GCN2)	240,212	266,028	(25,816)	111%	-11%
28	ERK1	181,820	202,357	(20,537)	111%	-11%
29	FAK	103,915	122,894	(18,979)	118%	-18%
30	FGFR1 (FLT2)	64,132	76,386	(12,254)	119%	-19%
31	GCK	326,016	315,596	10,420	97%	3%
32	GRK1	4,695	4,425	270	94%	6%
33	GSK3 beta	259,502	237,980	21,522	92%	8%
34	Haspin (GSG2)	649,776	567,600	82,176	87%	13%
35	HER2	17,086	16,024	1,062	94%	6%
36	HIPK1	220,703	225,947	(5,244)	102%	-2%
37	HPK1	505,596	458,118	47,478	91%	9%
38	IGF1R	111,183	130,972	(19,789)	118%	-18%
39	IKK alpha	23,730	23,944	(214)	101%	-1%
40	InsR	127,897	121,884	6,013	95%	5%

41	IRAK2	11,167	12,213	(1,046)	109%	-9%
42	JAK3	251,132	247,535	3,597	99%	1%
43	JNK1	260,451	281,079	(20,628)	108%	-8%
44	KDR	143,399	123,268	20,131	86%	14%
45	LCK	169,610	183,106	(13,496)	108%	-8%
46	LIMK1	9,169	9,837	(668)	107%	-7%
47	LRRK2	13,019	8,637	4,382	66%	34%
48	MAPKAPK2	684,637	663,505	21,132	97%	3%
49	MEK1	11,065	9,870	1,195	89%	11%
50	MEKK2	57,505	60,944	(3,439)	106%	-6%
51	MELK	196,694	190,938	5,756	97%	3%
52	MET	22,779	18,378	4,401	81%	19%
53	MLCK	77,724	81,195	(3,471)	104%	-4%
54	MNK1	145,676	124,582	21,094	86%	14%
55	MST1	129,380	139,489	(10,109)	108%	-8%
56	NDR	60,405	64,785	(4,380)	107%	-7%
57	NEK1	15,127	18,387	(3,260)	122%	-22%
58	NIK	20,768	18,639	2,129	90%	10%
59	p38 alpha	29,613	21,540	8,073	73%	27%
60	p70S6K	99,007	85,893	13,114	87%	13%
61	PAK1	13,614	7,852	5,762	58%	42%
62	PDGFR alpha	11,569	9,520	2,049	82%	18%
63	PDK1	38,399	32,272	6,127	84%	16%
64	PEAK1	48,788	47,952	836	98%	2%
65	PHKG1	34,809	27,513	7,296	79%	21%
66	PIM1	109,257	108,664	593	99%	1%
67	PKAc alpha	663,764	705,431	(41,667)	106%	-6%
68	PKC alpha	608,812	598,938	9,874	98%	2%
69	PKC beta I	233,612	228,387	5,225	98%	2%
70	PKC delta	103,168	122,939	(19,771)	119%	-19%
71	PKC mu	395,655	372,284	23,371	94%	6%
72	PKD2	239,936	191,560	48,376	80%	20%
73	PKN1/PRK1	2,048	1,319	729	64%	36%
74	PLK1	18,240	17,587	653	96%	4%
75	PRKG1	72,543	75,907	(3,364)	105%	-5%
76	RAF1(EE)	36,437	38,043	(1,606)	104%	-4%
77	RET	104,158	112,951	(8,793)	108%	-8%
78	RIPK1	83,489	65,885	17,604	79%	21%
79	ROR2	6,517	4,254	2,263	65%	35%
80	ROS1	224,185	241,677	(17,492)	108%	-8%
81	SBK1	80,555	73,208	7,347	91%	9%
82	SGK1	44,450	40,622	3,828	91%	9%
83	SIK	71,648	77,581	(5,933)	108%	-8%
84	SRC	80,755	23,783	56,972	29%	71%
85	STK19	14,557	12,624	1,933	87%	13%
86	STK3	47,152	54,332	(7,180)	115%	-15%
87	SYK	82,315	89,196	(6,881)	108%	-8%
88	TAK1-TAB1	45,160	48,799	(3,639)	108%	-8%
89	TBK1	222,624	201,825	20,799	91%	9%
90	TGFBR1 (ALK5)	24,198	9,472	14,726	39%	61%
91	TIE2	47,076	38,640	8,436	82%	18%
92	TLK1	509,942	447,043	62,899	88%	12%
93	TOPK	87,715	67,645	20,070	77%	23%
94	TRKA	124,500	125,192	(692)	101%	-1%
95	TSSK1B	166,193	155,343	10,850	93%	7%
96	TTK	25,004	25,840	(836)	103%	-3%
97	ULK1	145,631	158,046	(12,415)	109%	-9%
98	VRK1	9,162	9,109	53	99%	1%
99	WNK1	21,189	18,051	3,138	85%	15%
100	ZAK	155,102	153,022	2,080	99%	1%

Table S2: Activity change observed in the LKB1 Kinase tested against Activator-3 at 200μM concentration

Protein Kinase	Control (CPM)	Test (CPM)	% Change
LKB1	10984	11638	5%

Table S3: Evaluating the goodness of the representative structure obtained from MD simulations using PROCHECK server.

**Ramchandran Map**	**No. of resides**	**Percentage**
Most favored regions	889	85.8%
Additional allowed regions	128	12.4%
Generously allowed regions	13	1.3%
Disallowed regions	6	0.6%
Non-glycine and non-proline residues	1036	100.0%

Table S4: Heterotrimeric human AMPK crystal structures available in PDB.

**PDBID**	**Isoform**	**Organism**	**Inhibitor and/or activator**	**Modifications**	**References**
**5ISO**	α2β1γ1	Homo sapiens	AMP, Staurosporine, 992	108-SEP	NA
**4ZHX**	α2β1γ1	Homo sapiens	AMP, C2Z, C1V, Staurosporine,	172-TPO<br>108-SEP	Lagendorf et al. 2016[1]
**5EZV**	α2β1γ1	Homo sapiens	C2Z, C1V, Staurosporine	172-TPO<br>108-SEP	Lagendorf et al. 2016[1]
**4RER**	α1β2γ1	Homo sapiens	AMP, HEPES, B-	172-TPO	Li et al. 2015[2]

			cyclodextrin, Staurosporine	108-SEP	
**4REW**	α1β2γ1	Homo sapiens	AMP, Staurosporine	-	Li et al. 2015[2]
**4CFE**	α2β1γ1	Homo sapiens	AMP, Staurosporine, 991	172-TPO<br>108-SEP	Xiao et al. 2013[3]
**4CFF**	α2β1γ1	Homo sapiens	AMP, Staurosporine, A769662	172-TPO<br>108-SEP	Xiao et al. 2013[3]
**4CFH (2Y94)**	α1β2γ1	α1, γ1 -Rattus norvegicus<br>β2- Homo sapiens	AMP, Staurosporine	172-TPO	Xiao et al. 2011[4]

FigureS1

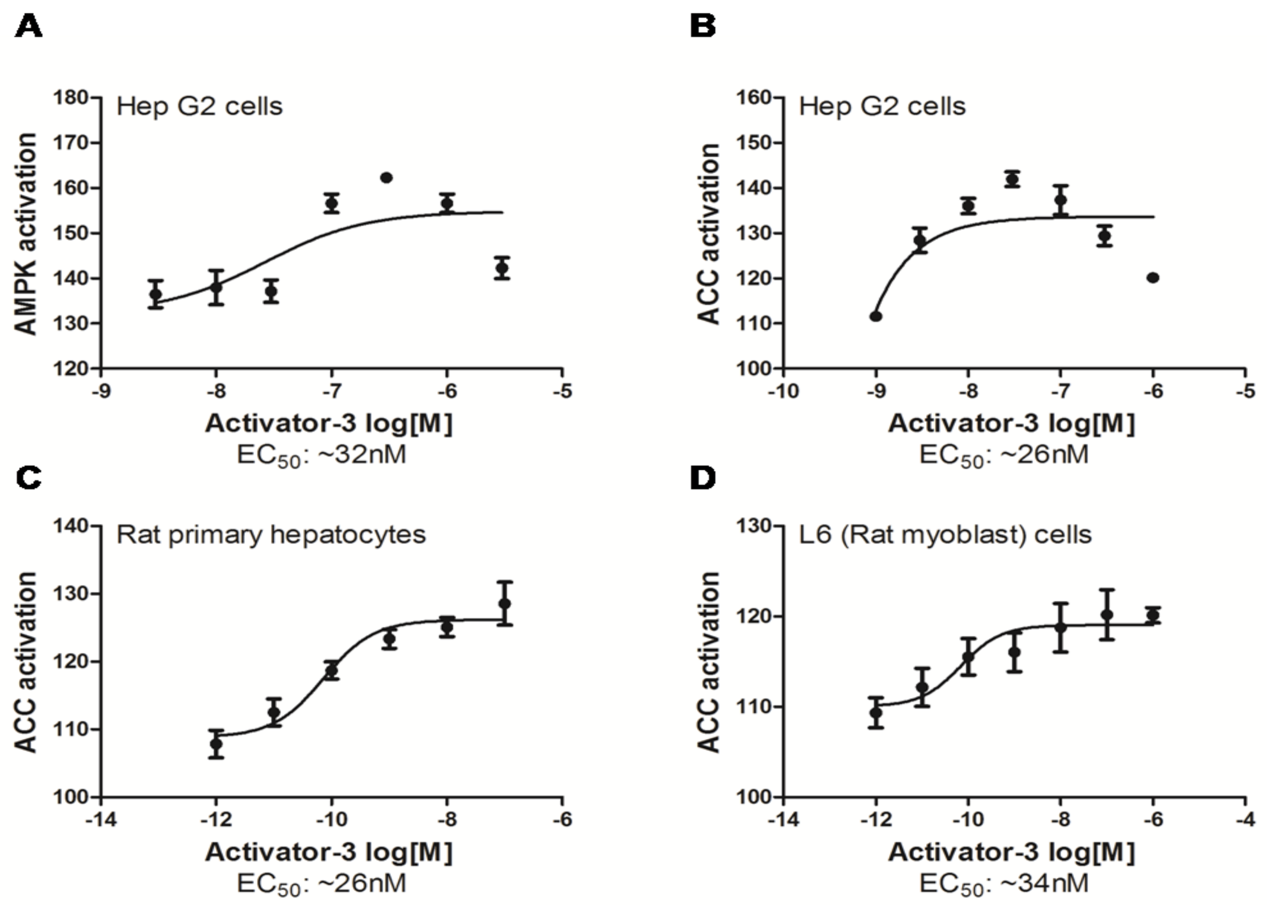

Figure S2

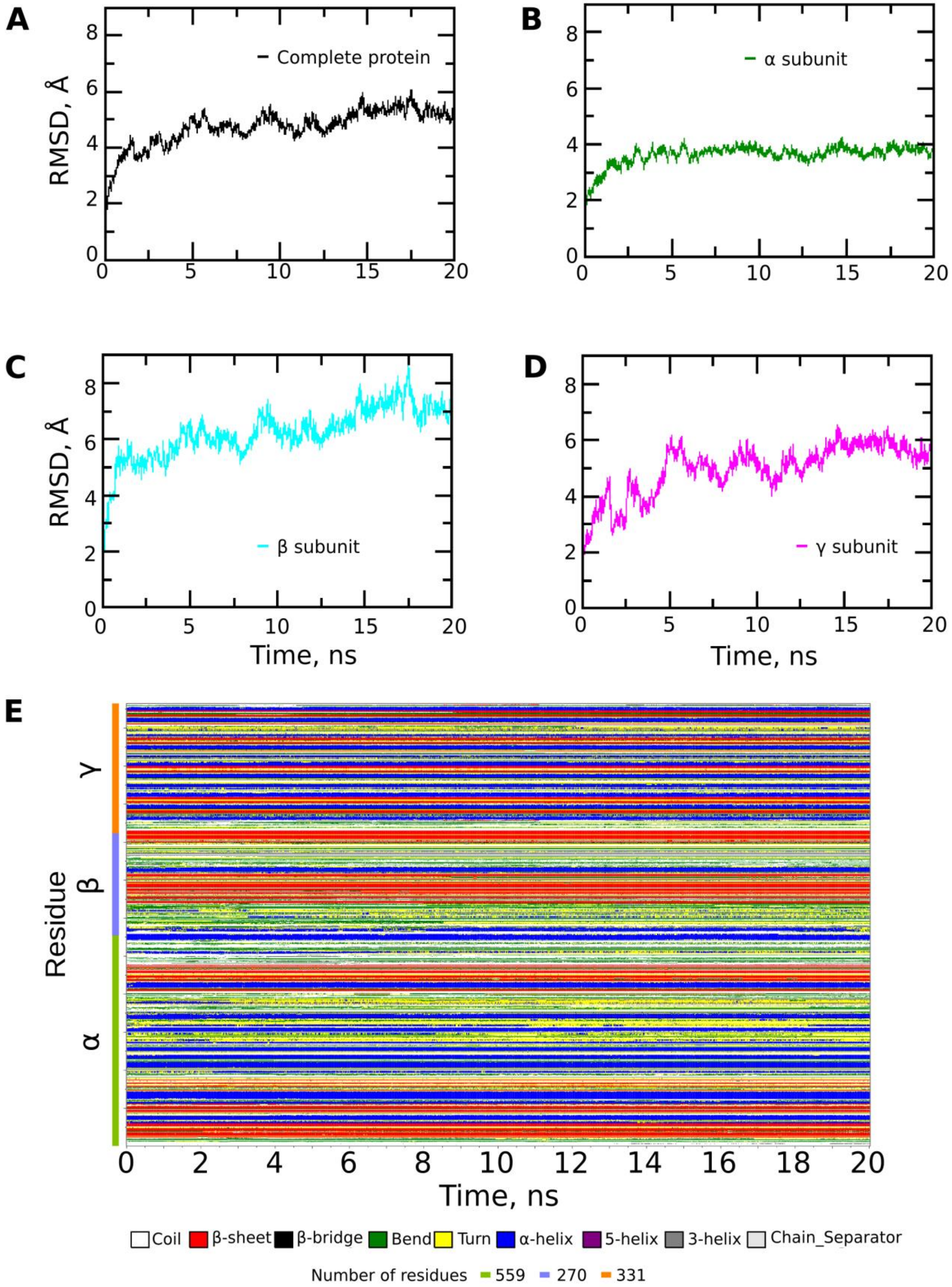
A
Complete protein
RMSD, Å
B
α subunit
C
β subunit
RMSD, Å
Time, ns
D
γ subunit
Time, ns
E
Residue
γ
β
α
Time, ns
Coil
β-sheet
β-bridge
Bend
Turn
α-helix
5-helix
3-helix
Chain_Separator
Number of residues
559
270
331

Figure S3

A

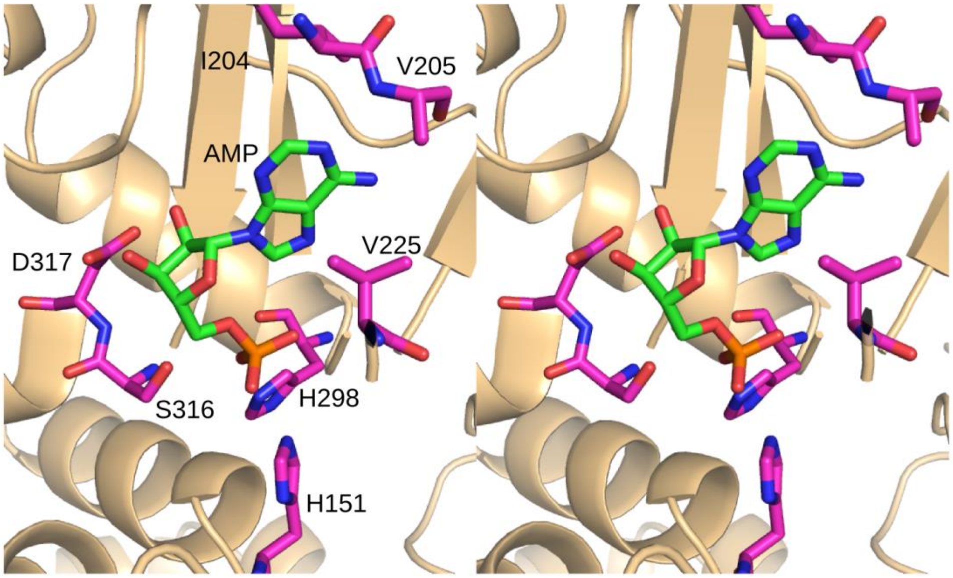
I204
V205
AMP
V225
D317
S316
H298
H151

B

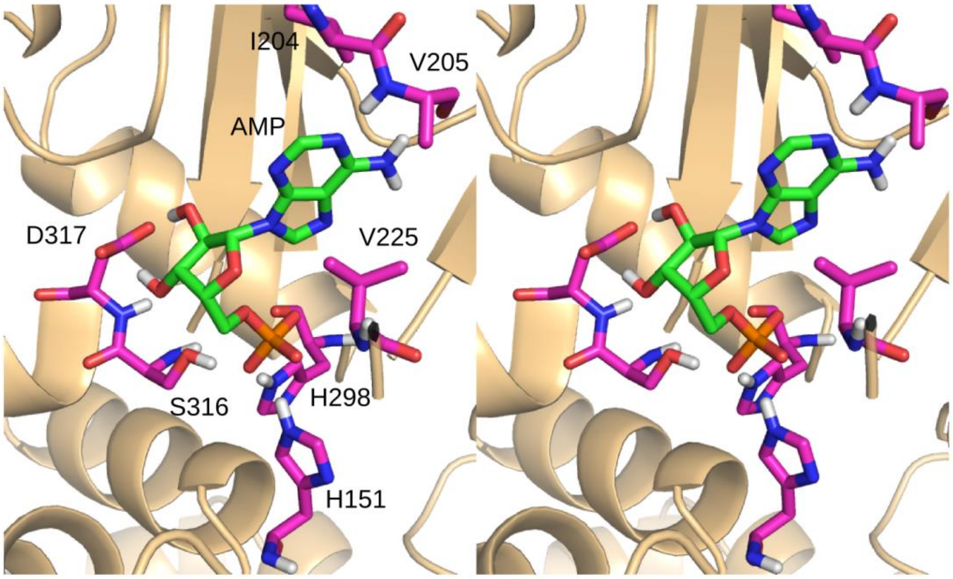
I204
V205
AMP
D317
V225
S316
H298
H151

Figure S4

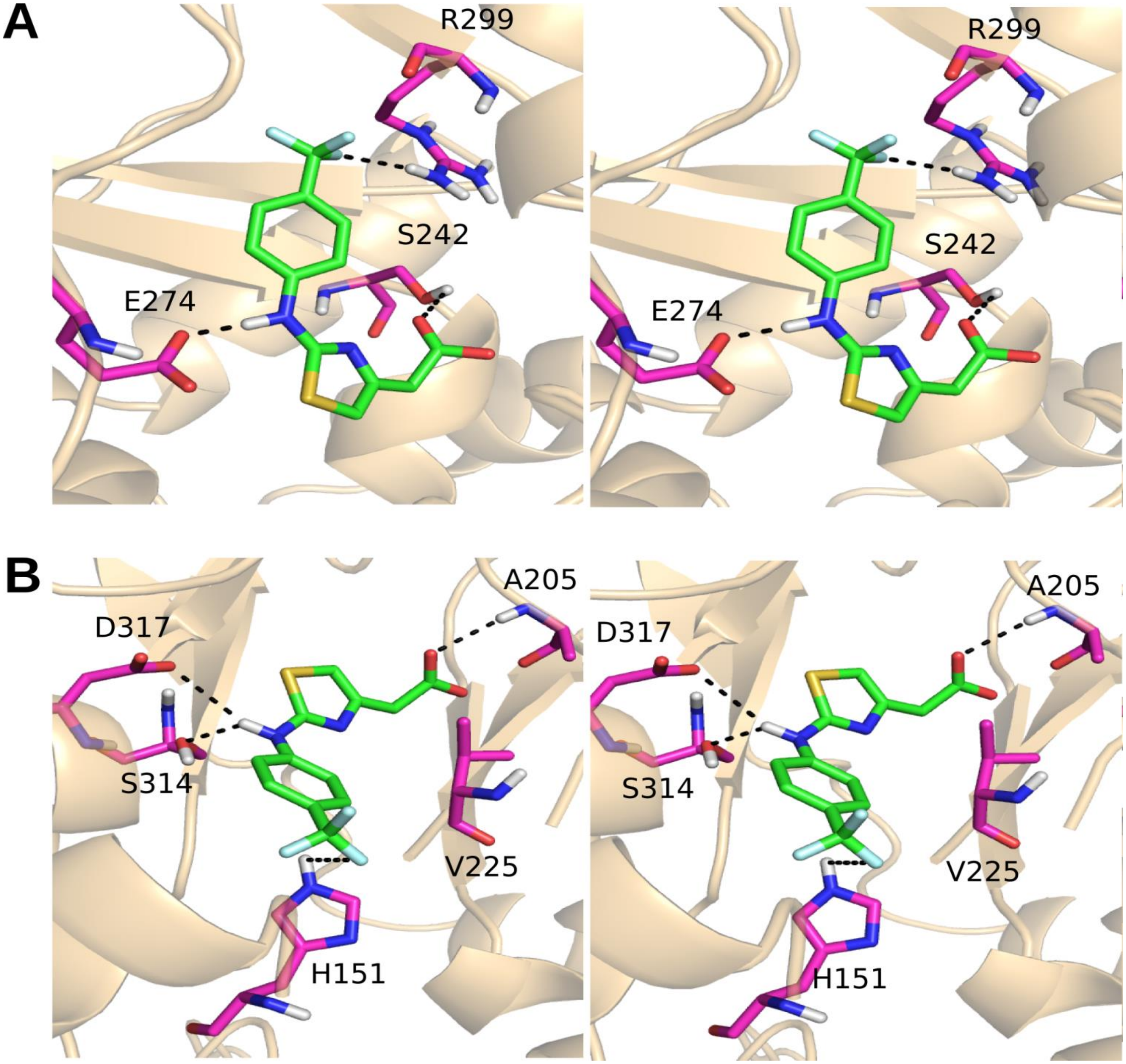
A
R299
S242
E274
R299
S242
E274
B
A205
D317
S314
V225
H151
A205
D317
S314
V225
H151

Figure S5

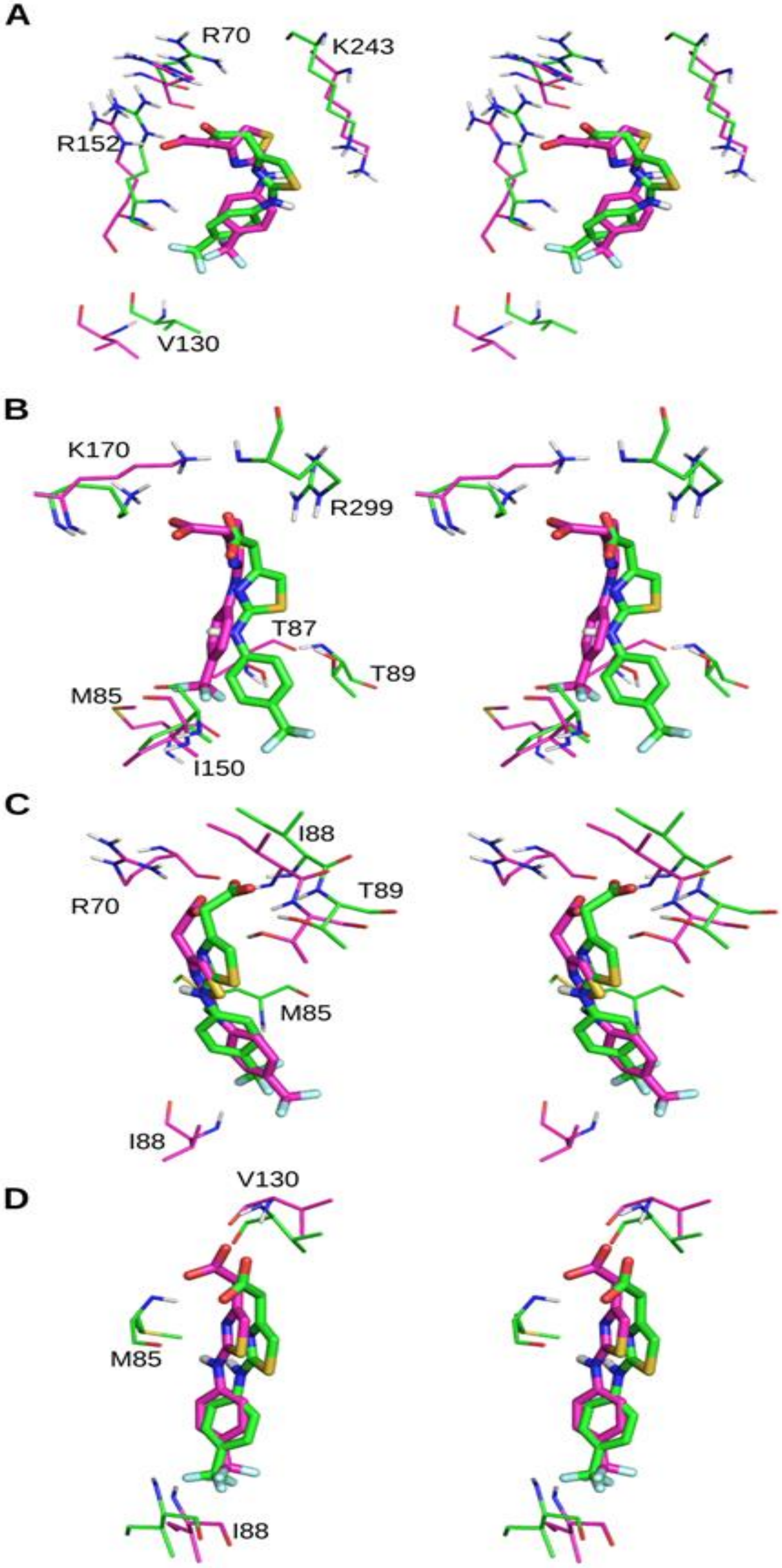
A
R70
K243
R152
V130
B
K170
R299
T87
T89
M85
I150
C
I88
T89
R70
M85
I88
D
V130
M85
I88

Figure S6

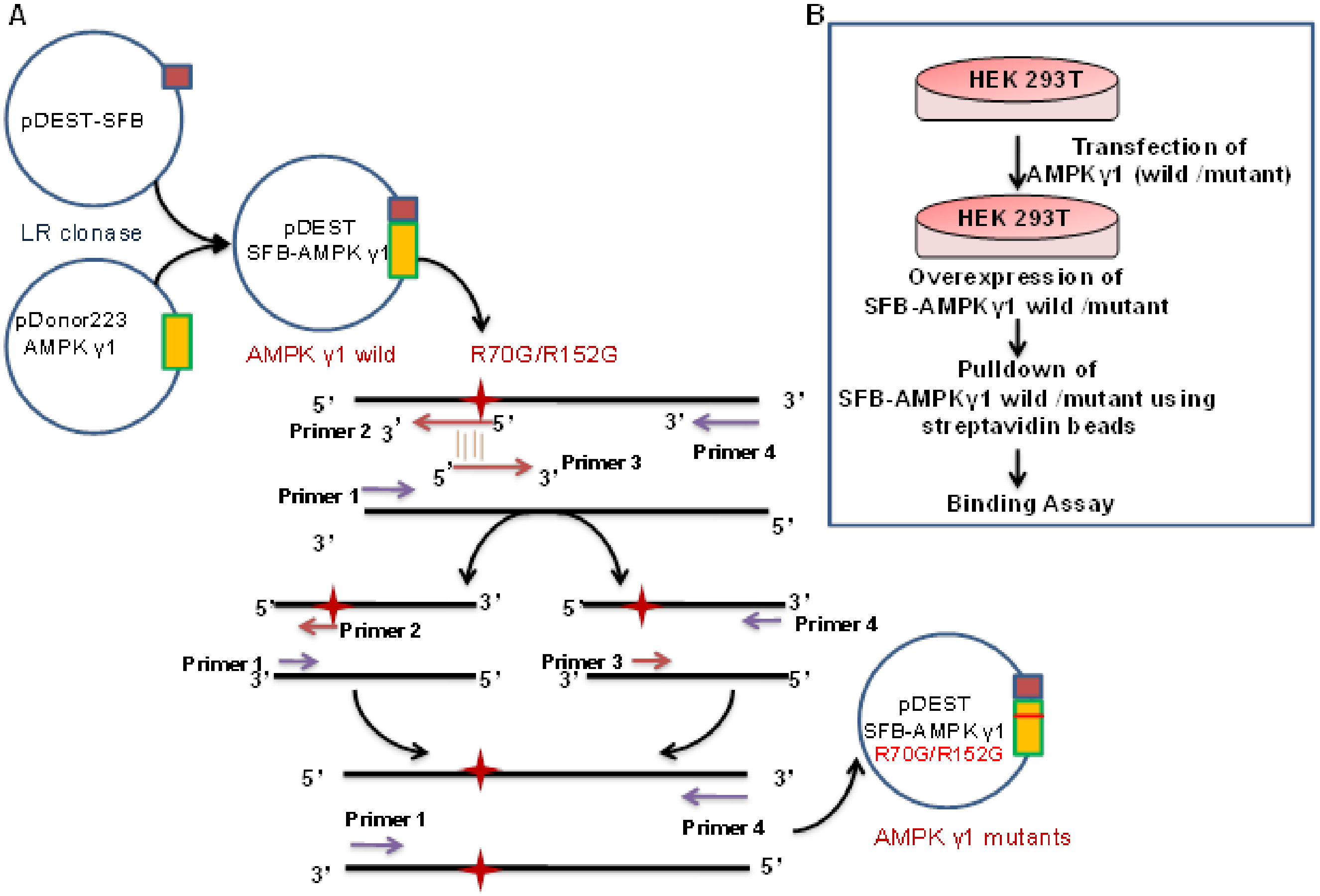
A
pDEST-SFB
LR clonase
pDonor223
AMPK γ1
pDEST
SFB-AMPK γ1
AMPK γ1 wild
R70G/R152G
5'
3'
Primer 2
Primer 3
Primer 4
Primer 1
pDEST
SFB-AMPK γ1
R70G/R152G
AMPK γ1 mutants
B
HEK 293T
Transfection of
AMPKγ1 (wild /mutant)
HEK 293T
Overexpression of
SFB-AMPKγ1 wild /mutant
Pulldown of
SFB-AMPKγ1 wild /mutant using
streptavidin beads
Binding Assay

Figure S7

	Pharmacokinetic profile in rat<br>Activator-3 (30mg/kg)		
	AUC (0-t) µg*h/ml	C_{max} (µg/ml)	$t_{1/2}$ (h)
Activator-3	978	109	4.11

- ❖Good PK profile
- ❖Bioavailability 100%
- ❖No significant BBB penetration
- ❖Molecular weight of Activator-3 = 302
- ❖AUC (978 µg*h/ml) = 3.3mM
- ❖C_{max} (109µg/ml) = 360µM

Figure S8

Full Blot of Fig. 2G

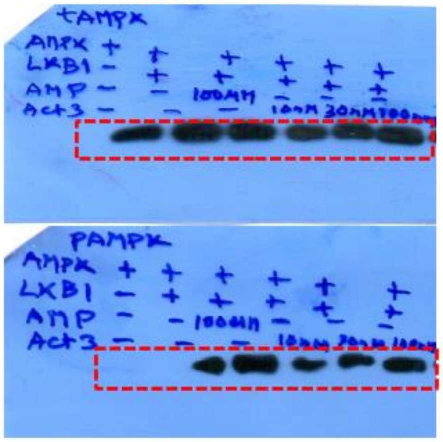

Full Blot of Fig. 2H

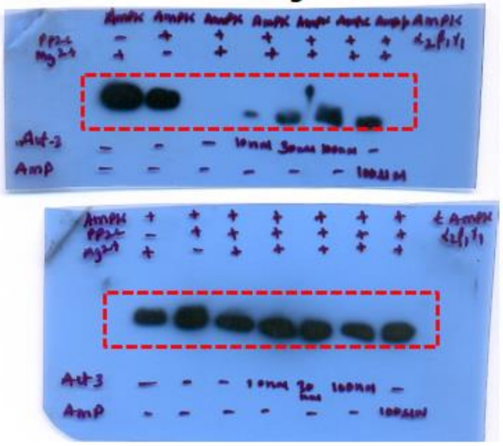

Full Blot of Fig. 6I

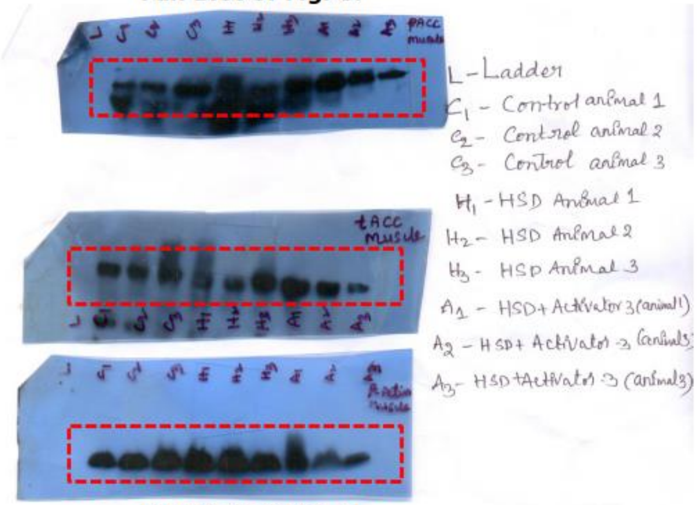

Full Blot of Fig. 6J

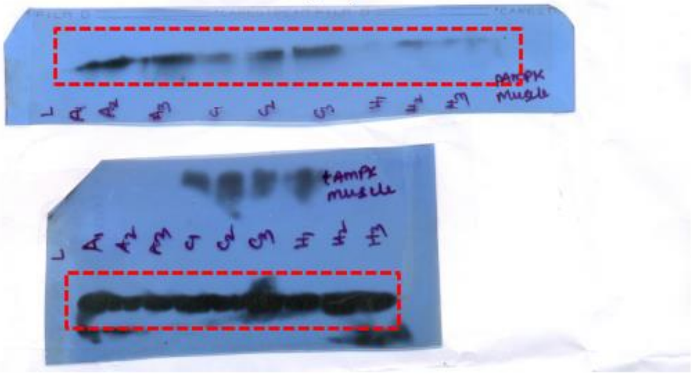

Figure S9:

ALPHA Subunit

```
AAPK1_HUMAN      1 MRRLSSWRKMATAEKQKHDGRVKIGHYILGDTLGVGTFGKVKVGKHELTG    50
4cfh_a.pdb       1 --------------------RVKIGHYILGDTLGVGTFGKVKVGKHELTG    30

AAPK1_HUMAN     51 HKVAVKILNRQKIRSLDVVGKIRREIQNLKLFRHPHIIKLYQVISTPSDI   100
4cfh_a.pdb      31 HKVAVKILNRQKIRSLDVVGKIRREIQNLKLFRHPHIIKLYQVISTPSDI    80

AAPK1_HUMAN    101 FMVMEYVSGGELFDYICKNGRLDEKESRRLFQQILSGVDYCHRHMVVHRD   150
4cfh_a.pdb      81 FMVMEYVSGGELFDYICKNGRLDEKESRRLFQQILSGVDYCHRHMVVHRD   130

AAPK1_HUMAN    151 LKPENVLLDAHMNAKIADFGLSNMMSDGEFLRTSCGSPNYAAPEVISGRL   200
4cfh_a.pdb     131 LKPENVLLDAHMNAKIADFGLSNMMSDGEFLRXSCGSPNYAAPEVISGRL   180

AAPK1_HUMAN    201 YAGPEVDIWSSGVILYALLCGTLPFDDDHVPTLFKKICDGIFYTPQYLNP   250
4cfh_a.pdb     181 YAGPEVDIWSSGVILYALLCGTLPFDDDHVPTLFKKICDGIFYTPQYLNP   230

AAPK1_HUMAN    251 SVISLLKHMLQVDPMKRATIKDIREHEWFKQDLPKYLFPEDPSYSSTMID   300
4cfh_a.pdb     231 SVISLLKHMLQVDPMKRATIKDIREHEWFKQDLPKYLFPED---------   271

AAPK1_HUMAN    301 DEALKEVCEKFECSEEEVLSCLYNRNHQDPLAVAYHLIIDNRRIMNEAKD   350
4cfh_a.pdb     272 -------------------------------AVAYHLIIDNRRIMNEAKD   290

AAPK1_HUMAN    351 FYLATSPPDSFLDDHHLTRPHPERVPFLVAETPRARHTLDELNPQKSKHQ   400
4cfh_a.pdb     291 FYLATSPPDSFLDDHHLTRPHPERVPFLVAETPRA---------------   325

AAPK1_HUMAN    401 GVRKAKWHLGIRSQSRPNDIMAEVCRAIKQLDYEWKVVNPYYLRVRRKNP   450
4cfh_a.pdb     326 ----AKWHLGIRSQSRPNDIMAEVCRAIKQLDYEWKVVNPYYLRVRRKNP   371

AAPK1_HUMAN    451 VTSTYSKMSLQLYQVDSRTYLLDFRSIDDEITEAKSGTATPQRSGSVSNY   500
4cfh_a.pdb     372 VTSTFSKMSLQLYQVDSRTYLLDFRSIDDEI-------------------   402

AAPK1_HUMAN    501 RSCQRSDSDAEAQGKSSEVSLTSSVTSLDSSPVDLTPRPGSHTIEFFEMC   550
4cfh_a.pdb     403 --------------------------------------------------   402

AAPK1_HUMAN    551 ANLIKILAQ     559
4cfh_a.pdb     403 ---------     402
```

Beta Subunit

```
AAKB1_HUMAN      1 MGNTSSERAALERHGGHKTPRRDSSGGTKDGDRPKILMDSPEDADLFHSE    50
4cff_b.pdb       1 --------------------------------------------------     0

AAKB1_HUMAN     51 EIKAPEKEEFLAWQHDLEVNDKAPAQARPTVFRWTGGGKEVYLSGSFNNW   100
4cff_b.pdb       1 ---------------------------RPTVFRWTGGGKEVYLSGSFNNW    23

AAKB1_HUMAN    101 SKLPLTRSHNNFVAILDLPEGEHQYKFFVDGQWTHDPSEPIVTSQLGTVN   150
4cff_b.pdb      24 SKLPLTRXHNNFVAILDLPEGEHQYKFFVDGQWTHDPSEPIVTSQLGTVN    73

AAKB1_HUMAN    151 NIIQVKKTDFEVFDALMVDSQKCSDVSELSSSPPGPYHQEPYVCKPEERF   200
4cff_b.pdb      74 NIIQVKKTDFEVFDALMVDSQKC-------------YHQEPYV-------   103

AAKB1_HUMAN    201 RAPPILPPHLLQVILNKDTGISCDPALLPEPNHVMLNHLYALSIKDGVMV   250
4cff_b.pdb     104 --PPILPPHLLQVILNKDTGISCDPALLPEPNHVMLNHLYALSIKDGVMV   151

AAKB1_HUMAN    251 LSATHRYKKKYVTTLLYKPI     270
4cff_b.pdb     152 LSATHRYKKKYVTTLLYKPI     171
```

Gamma Subunit

```
AAKG1_HUMAN      1 METVISSDSSPAVENEHPQETPESNNSVYTSFMKSHRCYDLIPTSSKLVV    50
4cff_e.pdb       1 --------------------------SVYTSFMKSHRCYDLIPTSSKLVV    24

AAKG1_HUMAN     51 FDTSLQVKKAFFALVTNGVRAAPLWDSKKQSFVGMLTITDFINILHRYYK   100
4cff_e.pdb      25 FDTSLQVKKAFFALVTNGVRAAPLWDSKKQSFVGMLTITDFINILHRYYK    74

AAKG1_HUMAN    101 SALVQIYELEEHKIETWREVYLQDSFKPLVCISPNASLFDAVSSLIRNKI   150
4cff_e.pdb      75 SALVQIYELEEHKIETWREVYLQDSFKPLVCISPNASLFDAVSSLIRNKI   124

AAKG1_HUMAN    151 HRLPVIDPESGNTLYILTHKRILKFLKLFITEFPKPEFMSKSLEELQIGT   200
4cff_e.pdb     125 HRLPVIDPESGNTLYILTHKRILKFLKLFITEFPKPEFMSKSLEELQIGT   174

AAKG1_HUMAN    201 YANIAMVRTTTPVYVALGIFVQHRVSALPVVDEKGRVVDIYSKFDVINLA   250
4cff_e.pdb     175 YANIAMVRTTTPVYVALGIFVQHRVSALPVVDEKGRVVDIYSKFDVINLA   224

AAKG1_HUMAN    251 AEKTYNNLDVSVTKALQHRSHYFEGVLKCYLHETLETIINRLVEAEVHRL   300
4cff_e.pdb     225 AEKTYNNLDVSVTKALQHRSHYFEGVLKCYLHETLETIINRLVEAEVHRL   274

AAKG1_HUMAN    301 VVVDENDVVKGIVSLSDILQALVLTGGEKKP     331
4cff_e.pdb     275 VVVDENDVVKGIVSLSDILQALVLT------     299
```

Legends of the Supplementary figures

Figure S1: Activator-3 activates AMPK and ACC in *in vitro* cell based assays**.** pAMPK (**A**) and pACC (**B, C and D**) based dose response curve of Activator-3 in HepG2 cells (**A,B)** primary Hepatocytes and L6 muscle cells (**C**,**D**).

Figure S2: Root mean square deviation (RMSD) of the protein backbone with respect to the energy minimized modeled structure for **A.** complete protein, **B.** α subunit, **C.** β subunit, **D.** γ subunit. **E.** DSSP plot showing secondary structure of the protein during the simulation. The color code for each of the secondary structure is given below the plot. α, β and γ subunits on the y-axis are demarcated using green, blue and magenta colors respectively.

Figure S3: Residues interacting with AMP molecule in the **A.** crystal structure (PDB: 4CFF) and **B.** Homology modeled structure. AMP and interacting residues are shown in green and magenta sticks respectively.

Figure S4: AMPK-activator-3 docked complexes for **A.** Site 3; **B**. Site 4. Activator-3 and interacting residues are shown in green and magenta sticks respectively.

Figure S5: Position of the Activator-3 before (green) and after 10ns MD simulation (magenta) in **A.** Wild-type; **B.** R70G mutant; **C.** R152G mutant; **D.** R70G&R152G (double) mutant

Figure S6: A. Schematic diagram of the constructs of human α1β1γ1 AMPK isoform and its mutants**. B.** Method of overexpression and purification of recombinant and its mutants human AMPK α1β1γ1 in HEK-293T cells used for enzyme assay.

Figure S7: Pharmacokinetics profile of Activator-3 in rats: HSD rats were treated with Activator-3 at 30mg/kg dose and different pharmacokinetics parameters were measured.

Figure S8: western blot raw data for pACC, total ACC, β-Actin, pAMPK and total AMPK for rat muscle tissue, LKB1 mediated phosphorylation of AMPK stimulated by Activator-3 and protection Assay using α2β1γ1.

Figure S9: Sequence alignment of α, β and γ subunits with the template structure that was used for modeling. The missing residues (pink) were modeled using either template based or ab initio modeling.

Supplementary references

1. Langendorf, C. G. *et al.* Structural basis of allosteric and synergistic activation of AMPK by furan-2-phosphonic derivative C2 binding. *Nat. Commun.* **7,** 10912 (2016).
2. Li, X. *et al.* Structural basis of AMPK regulation by adenine nucleotides and glycogen. *Cell Res.* **25,** 50–66 (2015).
3. Xiao, B. *et al.* Structural basis of AMPK regulation by small molecule activators. *Nat. Commun.* **4,** 3017 (2013).
4. Xiao, B. *et al.* Structure of mammalian AMPK and its regulation by ADP. *Nature* **472,** 230–233 (2011).
